# Precise Carboxylic Acid-Functionalized Polyesters in Reprocessable Vitrimers

**DOI:** 10.1021/jacs.4c14032

**Published:** 2025-02-14

**Authors:** Matilde Concilio, Gregory S. Sulley, Fernando Vidal, Steven Brown, Charlotte K. Williams

**Affiliations:** † Department of Chemistry, Chemistry Research Laboratory, University of Oxford, 12 Mansfield Road, Oxford OX1 3TA, U.K.; ‡ Scott Bader Company Ltd., Wellingborough Northamptonshire, Wollaston NN29 7RL, U.K.

## Abstract

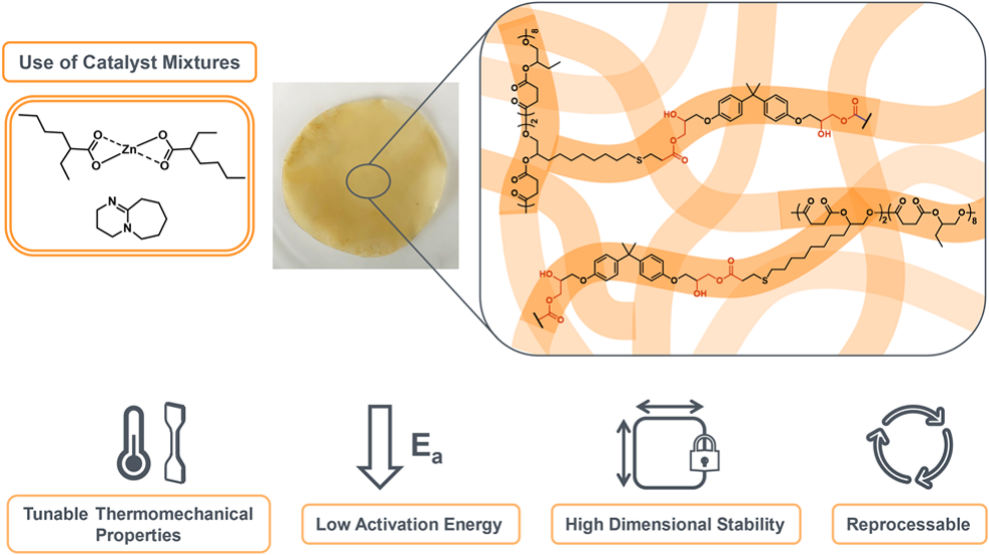

Thermosets are valued for their exceptional dimensional stability, mechanical properties, and resistance to creep and chemicals. Their permanent molecular structures limit reshaping, reprocessing, and recycling. Incorporating exchangeable chemical bonds into cross-linked polymer networks provides materials with thermoset-like properties that are also reprocessable. Here, ring-opening copolymerization (ROCOP) of unpurified, commercially available epoxides and succinic anhydride is employed to synthesize well-defined, low molecular weight polyesters with controlled functionalization. Polymer networks are then formed through the catalyzed reaction of these copolymers with the epoxy-containing cross-linker diglycidyl ether of bisphenol A. Catalyst mixtures of zinc bis(2-ethylhexanoate) and 1,8-diazabicyclo(5.4.0)undec-7-ene are used to assess the role of the catalysts in the curing and dynamic bond exchange reactions. Varying the catalyst ratios results in polymer networks with tunable mechanical properties (90% < ε_b_ < 450%, 0.30 MPa < UTS < 24 MPa), high creep recovery (%recovery > 90% after five creep cycles), and good reprocessability.

## Introduction

Epoxy resins have gained prominence in fiber-reinforced composites, engineering/structural adhesives, insulating materials, and high-performance coatings, due to their exceptional dimensional stability, mechanical properties, and resistance to both creep and chemicals.^1,2^ Nevertheless, their permanent molecular structure poses limitations, preventing them from being reshaped, manipulated, or effectively recycled. An intriguing chemical approach to introduce flexibility into cross-linked polymer networks involves the incorporation of exchangeable chemical bonds, which results in the formation of dynamic cross-links. These networks are known as covalent adaptable networks (CANs),^3^ and can be divided into two groups depending on the type of the dynamic bond exchange mechanism. In the case of CANs with a dissociative mechanism, bond breakage precedes bond formation, resulting in a different cross-linking density. For CANs with an associative exchange mechanism, bond-breaking and reformation occur simultaneously, therefore, a constant number of bonds is maintained throughout the exchange reactions. These materials are also characterized by an Arrhenius-like decrease in viscosity with temperature, a distinctive feature of vitreous silica, as shown for the first time by Leibler and co-workers in 2011.^4^ Consequently, this latter class of CANs is also referred as vitrimers.

Over the past decade, different chemistries have been explored for the synthesis of vitrimers. These include disulfide exchange,^5−12^ transamination of vinylogous urethanes,^13−19^ siloxane and silyl ether chemistries,^20−23^ olefin metathesis,^24,25^ imine chemistry,^26−30^ and dioxaborolane metathesis.^31−34^

These approaches are very interesting, but because they often require the synthesis of expensive or complicated monomers and cross-linkers, they are likely to be slower to translate to scale. Additionally, the resulting vitrimers can exhibit more limited mechanical properties (typically characterized by either low strain at break with high stress at break, or the inverse), and lack dimensional stability under common service temperature ranges (0–100 °C).

Therefore, it may be more effective to modify well-established and straightforward chemistries, such as transesterification, which can be easily implemented using commercially available monomers. For instance, transesterification between carboxylic acids and hydroxyl groups in the presence of a catalyst is one of the most used cross-linking reaction for the formation of vitrimers, as pioneered by Leibler and co-workers using epoxy- and COOH-containing compounds.^4,35^ In traditional epoxy/acid polymer networks, achieving sufficient free hydroxyl functions and carboxylic esters is ensured by the combination of bi- and poly functional monomers in various proportions. For these networks, the rate of transesterification is negligible at room temperature, but an increase in temperature, usually above 140 °C, allows a fast rearrangement of the polymer network, enabling the deformation, processing and recyclability of the material.^36^

The catalyst plays a crucial role by either promoting a favorable exchange pathway or lowering the barrier for the rate-determining step in the exchange reaction.^37^

This catalytic control should allow for tailoring the vitrimer’s viscous-flow behavior, as needed. However, selecting the appropriate catalyst often means striking a balance between facilitating rapid exchange and maintaining a sufficiently high energy barrier to ensure high exchange rates at processing temperatures, while significantly reducing or inhibiting them within the application temperature range.

Epoxy-vitrimers are defined by two key reactions: the initial curing or cross-linking reaction between epoxy and COOH functionalities, and the dynamic bond exchange reaction driven by transesterification. It has been demonstrated that both the type and amount of catalyst significantly influence the activation energy and exchange rate.^38^ Various catalysts have been employed to promote dynamic transesterification reactions, including Brønsted acids,^39^ Zn(OAc)_2_,^4^ organotin compounds, such as stannous octoate,^40^ dibutyltin dilaurate, dibutyltin diacetate, and dibutyltin bis(2,4-pentanedionate),^41^ triazabicyclodecene,^42^ and 1,8-diazabicyclo[5.4.0]undec-7-ene.^43^ Although it is assumed that the catalyst effectively promotes both reactions, it is crucial to establish this. Interestingly, the investigation of catalyst mixtures to enhance vitrimer properties has not yet been explored.

Here, catalyst mixtures of zinc bis(2-ethylhexanoate) (Zn(Oct)_2_), and 1,8-diazabicyclo(5.4.0)undec-7-ene (DBU) will be tested for both the epoxy cross-linking and the ester exchange reactions in polyester-based vitrimers. Well-defined, low molecular weight polyesters will be synthesized via ring opening copolymerization (ROCOP) of commercially available epoxides, namely 1,2-epoxy-5-hexene (vHO), 1,2-epoxy-9-decene (vDO), and 1,2-epoxybutane (BO), and succinic anhydride (SA). Postfunctionalization with carboxylic groups is expected to form polyesters with defined functionalization and low viscosity, ensuring good mixing with the epoxy cross-linker and catalysts. The resulting polymer networks are likely to exhibit high dimensional stability, which will be evaluated by measuring cyclic creep recovery at 50 °C. This temperature is above the glass transition temperature of the materials, and relevant for applications within the 0–100 °C range. They are also expected to have tunable mechanical properties, which will be assessed through tensile testing. Additionally, the polymer networks are anticipated to be reasonably reprocessable. This will be demonstrated by cutting the sample into pieces, reprocessing it through hot pressing, and then measuring the thermomechanical properties of the resulting material.

## Results and Discussion

### Polyester Production: SA/vHO Ring Opening Copolymerization

1,2-Epoxy-5-hexene and succinic anhydride were chosen for testing the suitability of the polymerization for industrialization (Scheme S1 and Table S2). The monomers were used as received, without any further purification steps. In the ROCOP, both a diol, 1,4-benzenedimethanol (BDM), and succinic acid, present as impurity in the starting anhydride (4.8 wt % from the ^1^H NMR spectrum, Figure S2), were independently evaluated as chain transfer agents (CTA). *tert*-Butylimino-tri(pyrrolidino)phosphorane (P1-tBu) was used as catalyst, and the reaction was carried out in toluene, at 100 °C, for 24 h. The final monomer concentration was 7.5 M, and the [cat]/[BDM]/[SA]/[vHO] ratio was kept constant to 1:16:200:300. The polymerization conducted using the succinic acid impurities as CTA was also performed both exposed to air, and with the addition of water (i.e., having both diacid and water as CTA). Additionally, the reaction was performed using a batch of SA containing only 0.9 wt % of diacid, and water was added as the only CTA (Table 1).

**Table 1:** Synthesis of vHO and SA Copolymers by Varying the Reaction Conditions^a^

sample	CTA	*M̅*_n,SEC_ (g mol^–1^)^c^	*D̵*^c^	diacid in SA (%)^d^
P1	diol	2700	1.22	4.8
P2	diacid	3000	1.16	4.8
P3^b^	diacid	2700	1.16	4.8
P4	diacid/water	3100	1.14	4.8
P5	water	3300	1.18	0.9

a [cat]/[CTA]/[SA]/[vHO] = 1:16:200:300, dry toluene, 100 °C, 24 h, under inert atmosphere.

b Reaction performed under air.

c *M̅*_n,SEC_ and *D̵* were measured by SEC (THF as eluent, 1 mL/min, 30 °C) calibrated using polystyrene standards.

d Measured by ^1^H NMR in DMSO-*d*_6_.by integration of the SA peak at 2.90 ppm and the diacid peak at 2.42 ppm.

**Figure 1:**
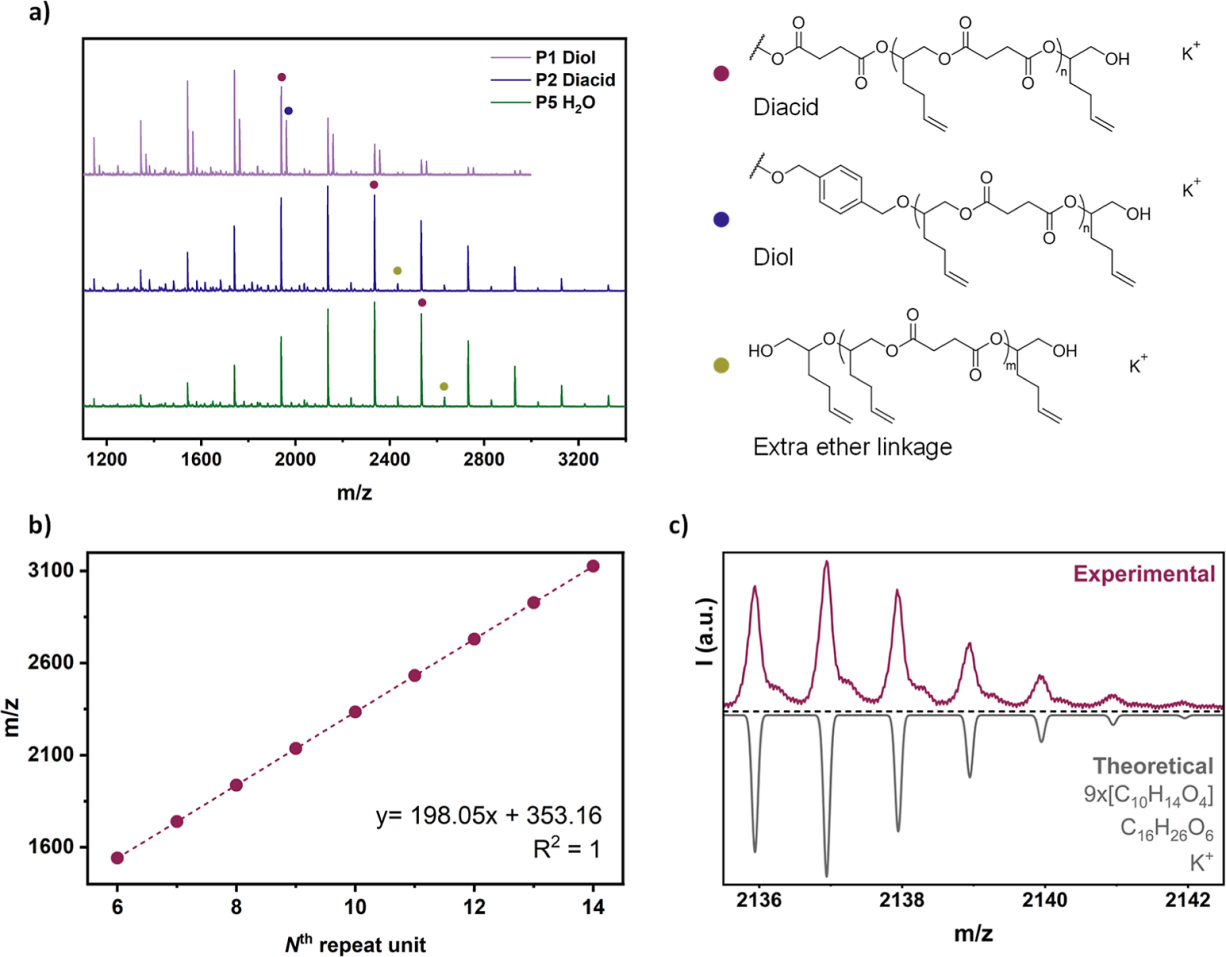
Characterization of vHO/BO copolymers. (a) MALDI-TOF spectra and main polymer structures for P1, P2 and P5 copolymers. (b) Plot of *m*/*z* vs N^th^ repeat unit. MW_theo_ of the N^th^ repeat unit (C_10_H_14_O_4_) = 198.21 g mol^–1^; MW_theo_ of the end group (C_16_H_26_O_6_) + K^+^ = 353.44 g mol^–1^. (c) Comparison of the experimental and theoretical isotope distributions for the peak at 2137 *m*/*z*, corresponding to the diacid-initiated 9th-mer.

The resulting polymers were characterized by ^1^H NMR spectroscopy (Figures S3–S7). They all exhibited monomodal molecular weight distributions with similar molecular weight values (*M̅*_n,SEC_ = 2700–3300 g mol^–1^) and narrow dispersity (*D̵* = 1.14–1.22, Figure S8). Furthermore, MALDI-TOF spectrometry was employed to study the polymer composition and end-groups (Figures 1a and S9). When the diol BDM was added as CTA, two main distributions were observed; the lowest intensity one corresponding to the BDM-initiated chains, and the highest intensity one to the polymers initiated from the succinic acid present in the anhydride. When diacid or water were used as CTAs, the main distribution corresponded to the diacid-initiated polymer chains terminated by OH groups, while a second distribution was attributed to the formation of chains with an additional ether linkage. It is important to note that in the MALDI spectra it is not possible to distinguish the diacid-initiated polymers from the polymers initiated by the reaction of water with an epoxide or anhydride, as the resulting chemical structures are identical. No additional distributions were observed, suggesting the absence of any side reactions. Moreover, the experimentally determined polymer repeat unit mass 198.05 g mol^–1^, obtained from the gradient of *m*/*z* vs N^th^ repeat unit, matched closely the theoretical value of 198.21 g mol^–1^ for SA/vHO (Figure 1b). Modeling of the isotopic distribution for the ninth-mer [molecular formula = (C_10_H_14_O_4_)_9_ for the polyester chain, C_16_H_26_O_6_ for the chain-end, and K^+^] exactly matched the experimental peaks centered at 2137 *m*/*z* (Figure 1c). Similar results were also obtained for the polymer chains with an extra ether linkage (Figure S10). These findings highlight the suitability of this prepolymer synthesis for subsequent larger scale (noting that reactions can be conducted without the toluene solvent, which would likely be preferable at scale).

**Table 2:** Synthesis of Copolymers Containing vHO, or vDO, and the Co-Epoxide BO

sample	epoxide	vHO/vDO^a^	BO^a^	*M̅*_n,SEC_ (g mol^–1^)^b^	*M̅*_n,th_ (g mol^–1^)	*D̵*^b^	*T*_g_ (°C)^c^	η_∞_ (Pa s)^d^
P2	vHO	1.0	0	3000	2600	1.16	–33	13
P2-COOH		1.0	0	3900	3900	1.07	–15	2000
P6	vDO	1.0	0	4000	3300	1.28	–55	3
P6-COOH		1.0	0	6800	4600	1.43	–26	300
P7	vHO/BO	0.20	0.80	2800	2300	1.09	–26	36
P7-COOH		0.20	0.80	3000	2600	1.14	–22	270
P8	vDO/BO	0.21	0.79	3300	2500	1.13	–33	16
P8-COOH		0.21	0.79	4000	2700	1.14	–27	111

a Calculated from the ^1^H NMR spectra in CDCl_3_ of the unfunctionalized copolymers by integration of the peaks at 0.90 ppm corresponding to the 3H of the BO methyl group, and at 5.75 ppm (or 5.78 ppm) corresponding to the single proton of the vHO (or vDO) double bond (see Figures S11 and S12 in Supporting Information).

b *M̅*_n,SEC_ and D̵ were measured by SEC (THF as eluent, 1 mL min^–1^, 35 °C).

c Obtained from the DSC data of the second heating cycle.

d Shear viscosity measured at 30 °C using oscillatory shear rheology (see Supporting Information).

### Low Viscosity Polyesters via Ring Opening Copolymerization

**Scheme 1:**
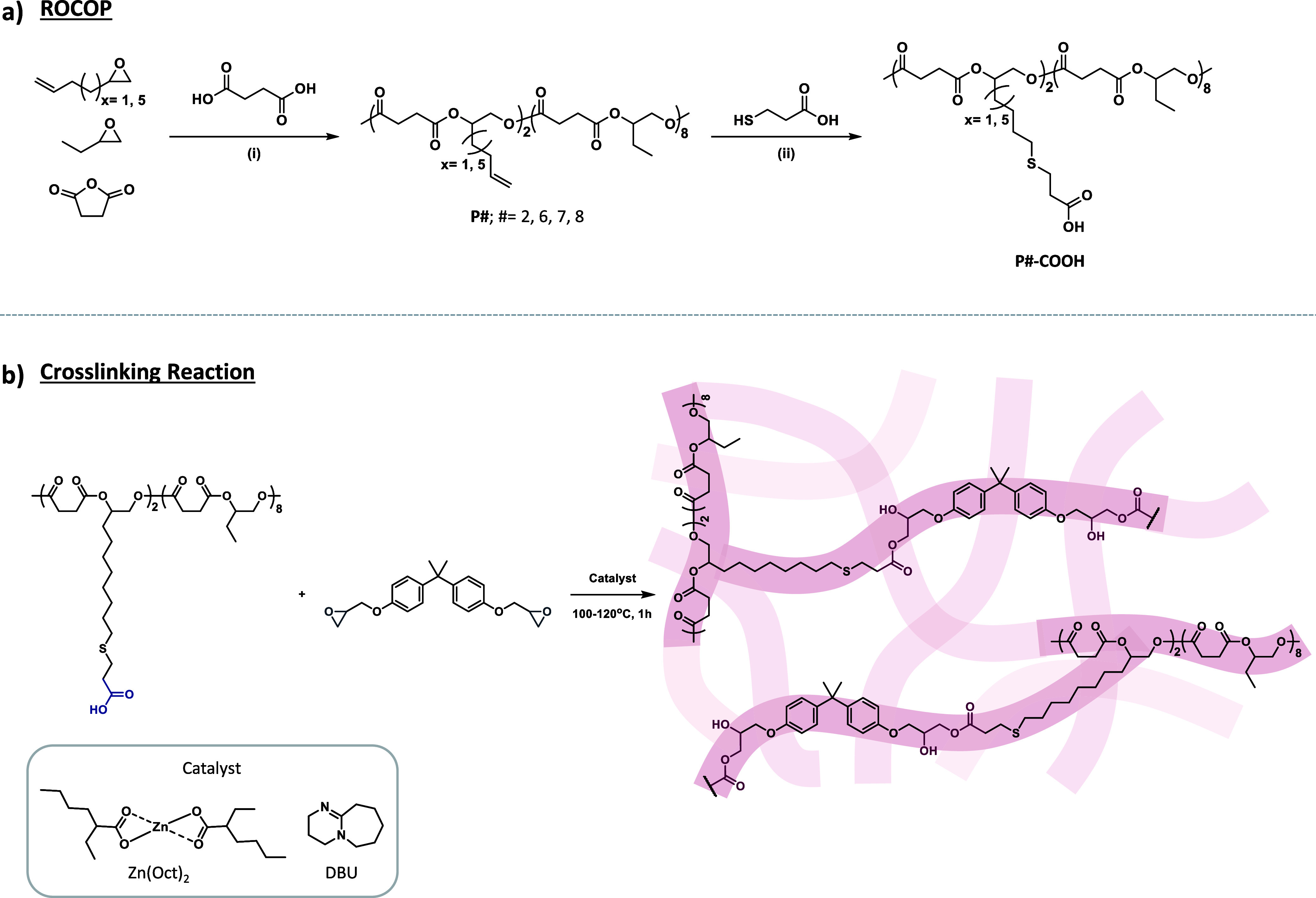
Synthesis of the Epoxide/SA Polyesters and of the Polyester-Based Networks Reaction scheme of the ROCOP of SA with vHO (*x* = 1) or vDO (*x* = 5) and the coepoxide BO (P#, # = 2, 6, 7, 8); followed by COOH functionalization via thiol–ene reaction (P#-COOH). (i) [cat]/[CTA]/[SA]/[epoxides] = 1:16:200:300, vHO (or vDO):BO = 20:80, dry toluene, 100 °C, 24 h. (ii) [DMPA]/[alkene]/[thiol] = 0.2:1:2, dry THF, RT, 1 h, UV. Network formation via cross-linking reaction between the vDO/BO/SA copolymer and DGEBA in the presence of Zn(Oct)_2_ or DBU as catalyst. [COOH]/[epoxy]/[cat] = 1:1:0.05, 100–120 °C, 1 h.

A low prepolymer viscosity is essential to facilitate mixing with the other compounds for the formation of the networks. Copolymerizations conducted with a second monomer to disrupt both inter- and intramolecular interactions between functional groups, or increasing the length of the alkyl side chains, could enhance overall chain mobility and thereby reduce the overall polymer viscosity. Four copolymers of SA with vHO (**P2**) or 1,2-epoxy-9-decene (vDO, **P6**) and the copolymers vHO/BO and vDO/BO (**P7** and **P8**, respectively) were synthesized via ROCOP (Scheme 1a, Table 2, Figures S4, S11–S13). The copolymerizations were conducted utilizing a [cat]/[CTA]/[SA]/[epoxides] ratio of 1:16:200:300, with a vHO (or vDO) to BO ratio of 20:80 (i.e., 20% functionalization), and succinic acid as the CTA. The concentration of diacid was adjusted considering the amount of succinic acid already present in the specific batch of anhydride used. The composition of the vHO/BO/SA and vDO/BO/SA copolymers was determined, from the ^1^H NMR spectra by integration of the peaks at 0.90 ppm corresponding to the 3H of the BO methyl group, and at 5.75 ppm (or 5.78 ppm) corresponding to the single proton of the vHO (or vDO) double bond. The spectra showed that the epoxides were randomly distributed along the polymer chain and had comparable reactivity, since both epoxides were used in excess and the experimental molar ratios matched the theoretical ones.

Furthermore, the synthesis of copolymers rather than two single homopolymers was further validated through Diffusion Ordered Spectroscopy (DOSY), where only one signal corresponding to the copolymer was observed (Figure S14). All copolymers were then functionalized via thiol–ene reaction using 3-mercaptopropionic acid, in the presence of 2,2-dimethoxy-2-phenylacetophenone (DMPA) photoinitiator ([alkene]/[thiol]/[DMPA] = 1:2:0.2), in THF, at a polymer concentration of 100 mg mL^–1^. The reaction was performed at room temperature, for 1 h, under UV irradiation. Full alkene functionalization was confirmed by ^1^H NMR spectroscopy for all copolymers, as demonstrated by the disappearance of the alkene peaks in the 5.0–6.0 ppm area and the appearance of the thiol peaks in the range 2.5–3.0 ppm (Figures S15–S18). Each copolymer possessed an average of two carboxylic groups per chain, corresponding to a degree of functionalization of 20%. The success of the thiol–ene reaction was further confirmed by a shift in the molecular weight toward higher values (Figure S19), while maintaining monomodal distributions and low dispersity (*D̵* < 1.14). From SEC analysis, P6 exhibited a broader molecular weight distribution both before and after functionalization compared to the other copolymers. This was attributed to the higher number of long alkyl chains and COOH functionalities, potentially enhancing the interaction with the SEC column and causing a change in the hydrodynamic volume of the polymer.

All functionalized polyesters showed thermal stability, in air, above 280 °C (Figure S20 and Table S4). DSC analysis revealed a single thermal transition corresponding to a glass transition, with the carboxylic acid-functionalized copolymers having higher glass transition temperature (*T*_g_) values compared to their alkene-containing counterparts. A higher *T*_g_ was observed in the copolymers of SA with vHO or vDO, showing increases of +18 and +29 °C, respectively (Figure S21). The copolymerization with a second epoxide reduced the difference in *T*_g_ to just +5 °C, with the vDO/BO/SA copolymer **P8-COOH** showing the lowest *T*_g_ at −27 °C (Figure S22). This increase in *T*_g_ was attributed to the formation of inter- and intramolecular hydrogen bonding interactions between the carboxylic groups present on the polymer chains. The formation of hydrogen bonds also led to a significant rise in viscosity between the alkene-containing and the fully functionalized polyesters, as shown by the shear viscosity values at 30 °C (Figure S23). The vHO/SA copolymers exhibited the greatest increase, with viscosity rising from 13 to 2000 Pa s for **P2** and **P2-COOH**, respectively. In contrast, the vDO/BO/SA copolymers, **P8** and **P8-COOH**, showed the lowest increase, with viscosity rising from 16 to 111 Pa s. Therefore, **P8-COOH** was selected for the formation of the networks due to its lowest viscosity after functionalization.

### Polyester Networks

The networks were synthesized from diglycidyl ether of bisphenol A (DGEBA), a major component of epoxy resins and a widely used cross-linker, and the COOH-functionalized vDO/BO/SA copolymer **P8-COOH** containing an average of two carboxylic acids per chain (Scheme 1b). The **P8-COOH** copolymer also had hydroxyl end groups. However, because epoxy groups react more quickly with carboxylic acids than with OH groups, this latter reaction can be considered negligible.^44^ Due to the susceptibility of the epoxy–acid reaction to side reactions,^45^ a catalyst is typically used to promote the opening of the epoxy ring.^46^ The stoichiometry was kept to one carboxylic acid per epoxy function (COOH/epoxy = 1:1) to promote transesterification.^47^ Two catalysts were tested: the Lewis acid zinc bis(2-ethylhexanoate) (Zn(Oct)_2_), and the base 1,8-diazabicyclo(5.4.0)undec-7-ene (DBU), which are widely recognized for their ability to accelerate the hydroxyl ester exchange between epoxy glycidyl ethers and carboxylic acids.^35,40,48−50^ Both catalysts were tested at a concentration of [COOH]/[cat] = 1:0.05.

It is worth mentioning that for the network synthesis, polymers with different degrees of COOH functionalization, 100% and 50%, were also evaluated (**P_vDO100%_** and **P_vDO50%_** in Table S2). Both polymers, when cured in the presence of DGEBA and Zn(Oct)_2_ ([COOH]/[epoxy]/[cat] = 1:1:0.05), produced extremely brittle films (Figure S24). This brittleness was attributed to the limited mobility of polymer chains caused by the high cross-linking density. Furthermore, a 20% hydroxyl-functionalized polymer (**P8-OH**) was also tested. However, the obtained network did not exhibit stress-relaxation behavior (Figure S25). Therefore, the **P8-COOH** copolymer was selected as the best candidate for the network synthesis.

To select the appropriate curing temperature, the network formation was studied using DSC and rheological measurements (Figure S26). Regardless of the catalyst used, the resulting curing reactions were complex, exhibiting multiple exothermic peaks, with two major events occurring at ∼100 and >140 °C (Figure S26a). By rheological analyses, the storage (*G*′) and loss moduli (*G*″) were measured as a function of temperature and the gelation temperature (*T*_gel_) was determined at the moduli crossover point (Figure S26b). The two catalysts exhibited similar *T*_gel_ values, with Zn(Oct)_2_ at 101 °C and DBU at 109 °C.

Although the formation of a network from the polyaddition reaction of diepoxy and diacid compounds might be somewhat challenging to envision, since this reaction might be expected to yield linear products with theoretically infinite molar masses by controlling stoichiometry, the gel point of the curing reaction would not be reached if polyaddition were the only reaction taking place. However, rheological studies of the two curing reactions revealed the presence of a gel point. It has been demonstrated that in catalyzed stoichiometric systems, transesterification occurs following addition esterification, resulting in branching points and, ultimately, network formation.^51,52^ To gain deeper insights into the curing process, network formation using the two different catalysts was investigated by performing rheological isothermal experiments, at 120 and 150 °C, for 1 h, measuring the time required to reach the moduli crossover point. When Zn(Oct)_2_ was used as the catalyst, no moduli crossover was observed at 120 °C after 1 h (Figure S27a), indicating only the formation of long polymer chains via the addition esterification of epoxy and acid functionalities. However, at 150 °C, a gel point was quickly reached within 5 min (Figure S27b), suggesting that transesterification reactions had occurred, leading to network formation. In contrast, when DBU was used as the catalyst, a gel point was reached at 120 °C within just 1 min (Figure S28), indicating that this base more effectively catalyzed both the polyaddition and the subsequent transesterification reactions. At the end of the curing reactions, the two networks exhibited different *G*′ values, indicating variations in the degree of transesterification. The network obtained using the Zn catalyst showed a lower *G*′ value (∼0.05 MPa), while the network formed with DBU exhibited a higher *G*′ (∼2.95 MPa). This difference suggests a variation in the extent of the transesterification reaction and, consequently, in the cross-linking density between the two networks. It has been proposed that the mechanism of transesterification catalysis differs depending on whether a metal catalyst or an organic base is used.^53^ Metal catalysts are proposed to remain bound to the network, with transesterification occurring only when both the alcohol and ester groups are present within the coordination sphere of the metal.^53^ In contrast, organic bases, which are hypothesized to be uncoordinated in the network, are more mobile and can diffuse freely, facilitating transesterification by interacting with multiple exchange sites.^53^

### Characterization of the Polyester-Based Networks

**Figure 2:**
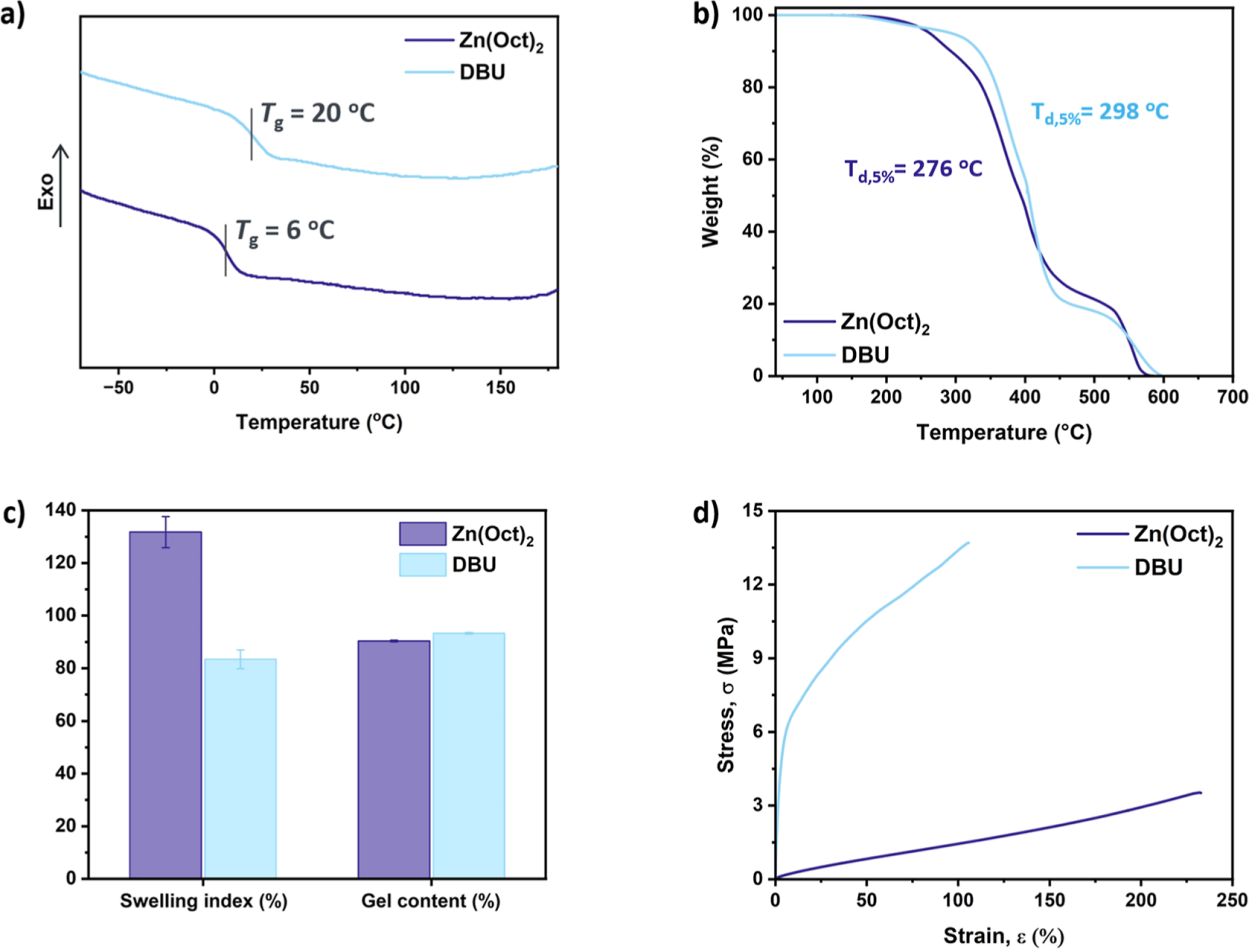
Characterization of the cross-linked networks based on P8-COOH/DGEBA/Zn(Oct)_2_ or DBU. (a) DSC data of the first heating cycle (exo up, normalized to the same heat flow per gram). (b) Thermogravimetric curves under air. (c) Swelling index and gel content measured after swelling the polymer networks in THF for 24 h and drying for 96 h. (d) Representative stress–strain curves of the cured materials obtained using Zn(Oct)_2_ (dark blue), or DBU (light blue).

To form the polymer networks, the two reaction mixtures containing P8-COOH, DGEBA, and the catalyst (Zn(Oct)_2_ or DBU) were first partially cured in a vacuum oven, at 120 °C for 1 h, and then compression molded, at 150–170 °C for 1 h. DSC and DMA analyses revealed fully cross-linked materials having only a glass transition with *T*_g_ values at 6 and 20 °C for the Zn(Oct)_2_- and DBU-containing samples, respectively (Figures 2a, S29 and Table S5). The DMA data also revealed that the DBU-containing network exhibited a higher storage modulus (*E*′) in the rubbery plateau (*E*′ = 7 MPa) compared to the Zn(Oct)_2_ network (2 MPa), indicating a higher cross-linking density. The polymer networks also demonstrated high thermal stability above 260 °C, with similar degradation profiles (Figure 2b). Swelling experiments performed in THF confirmed gel contents above 90% for both samples, but different swelling indices indicated a different cross-linking density (Figure 2c). The data confirmed that DBU is a more effective catalyst for transesterification compared to Zn(Oct)_2_. This is evidenced by a lower swelling index of 83 ± 4% and, consequently, a higher cross-linking density compared to the Zn(Oct)_2_ network having a swelling index of 132 ± 6%. These results are in agreement with the DSC and rheological data, where a higher *T*_g_ and *G*′, indicative of a higher cross-linking density, were measured for the DBU-containing sample (*T*_g_ = 20 °C and *G*′ = 2.95 MPa).

Uniaxial extension experiments were performed according to ISO 527 on dumbbell-shaped specimens (ISO 527-2 type 5B, five technical replicates). The data showed that the sample with a higher cross-linking density exhibited more plastic-like behavior, characterized by a greater Young’s modulus (*E* ∼ 288 MPa), ultimate tensile strength (UTS ∼ 14 MPa), and toughness (∼11 MPa), along with a reduced strain at break (ε_b_ ∼ 108%). In contrast, the material containing Zn(Oct)_2_ displayed more elastomeric-like behavior, with a lower Young’s modulus (*E* ∼ 2 MPa), ultimate tensile strength (UTS ∼ 3 MPa), toughness (∼4 MPa), and a higher strain at break (ε_b_ ∼ 238%, Figure 2d, Table S6).

### Dynamic Bond Exchange in the Polyester-Based Networks

**Figure 3:**
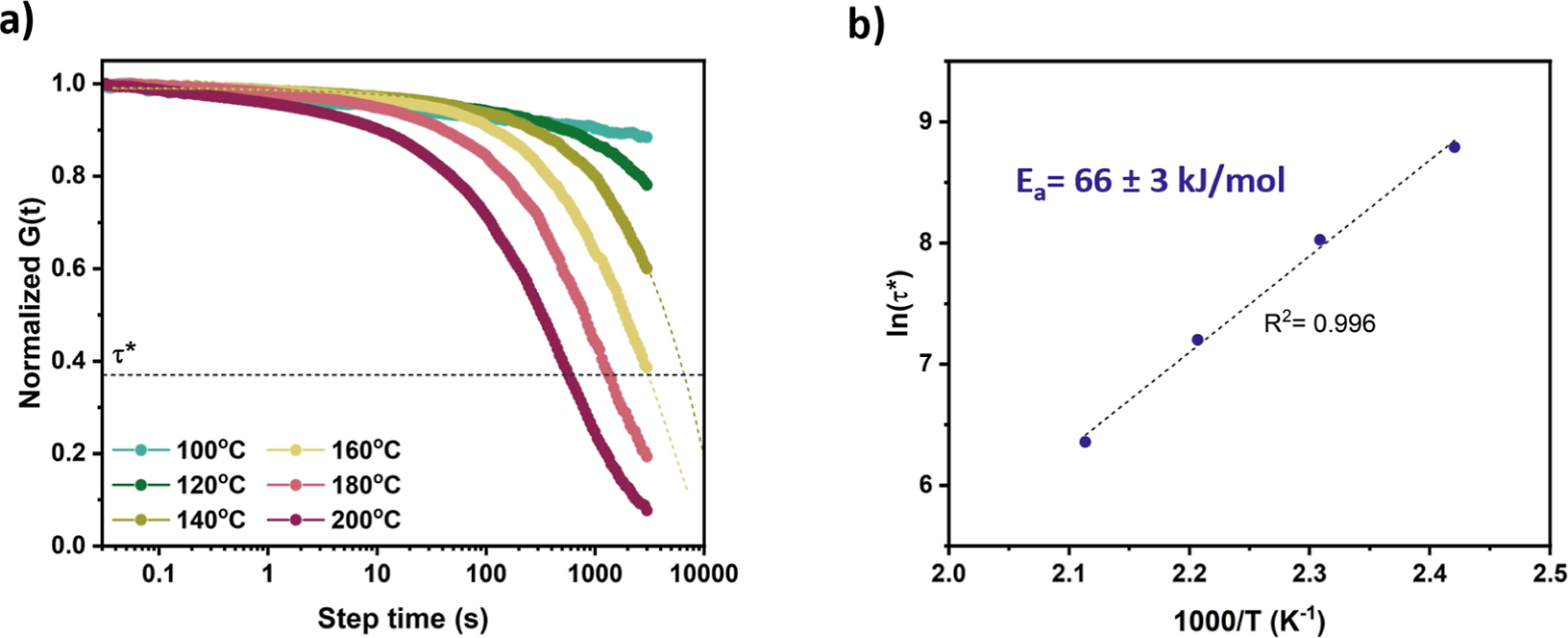
Dynamic bond exchange in the P8-COOH/DGEBA/Zn(Oct)_2_ vitrimer. (a) Normalized stress relaxation data, and (b) plot of ln(τ*) vs 1000/*T* with a linear fit to extract the activation energy.

Vitrimers show that the stress caused by deformation can be released at high temperatures through dynamic cross-linking exchange. To establish whether the network behaved as a vitrimer, stress-relaxation experiments were performed between 100 and 200 °C on the two samples. In these experiments, *G*(*t*) was measured as a function of time at each temperature. The DBU-containing network exhibited a decrease in relaxation time as the temperature increased from 100 to 140 °C (Figure S30). However, above 150 °C, the relaxation time began to increase, indicating that additional transesterification reactions occurred, resulting in increased cross-linking density and, consequently, longer relaxation times. On the contrary, the Zn(Oct)_2_-containing sample showed relaxation times decreasing with increasing temperature (Figure 3a). Based on the Maxwell model for viscoelastic fluids, relaxation time values (τ*) were obtained at 0.37 (1/*e*) of the normalized *G*(*t*). The activation energy was extrapolated by plotting the ln(τ*) values as a function of 1000/*T* (Figure 3b). The linear trend observed is a characteristic feature of vitrimeric materials.^39^ Using the Arrhenius rate equation (eq 1), where τ_0_ is the relaxation time at infinite temperature, *R* is the universal gas constant, and *T* is the absolute temperature, an activation energy (*E*_a_) for the dynamic bond exchange of 66 ± 3 kJ mol^–1^ was determined.
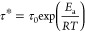
1

This value aligns with the activation energy measured for other transesterification-based vitrimers, where values typically occur within the range of 68–129 kJ mol^–1^.^35,54−58^ These results suggest that the choice of catalyst plays a critical role in tuning the thermomechanical properties and stress relaxation behavior of the dynamic networks. DBU demonstrated superior catalytic activity for the transesterification reaction, resulting in a more highly cross-linked network with better mechanical properties. In contrast, Zn(Oct)_2_ produced a less cross-linked network, which was better able to dissipate applied stress as the temperature increased. Therefore, various Zn(Oct)_2_/DBU ratios were evaluated to identify the most effective catalyst mixture(s), to achieve complete and rapid cross-linking, while facilitating temperature-dependent exchange reactions.

### Use of Zn(Oct)_2_ and DBU Catalyst Mixtures

A series of mixed Zn(Oct)_2_/DBU catalyst combinations were tested to make the vitrimers. In all cases, the overall catalyst concentration was kept to [COOH]/[cat] = 1:0.05. The curing processes were first monitored via DSC and rheological analysis. The exothermic phenomena in the DSC curves shifted to higher temperatures with increasing content of Zn(Oct)_2_ (Figure S31). A similar trend was also observed in the rheological data, where a rise in *T*_gel_ was detected with increasing the Zn catalyst content (Figure S32).

The large-scale polymer networks were synthesized by partially curing the reaction mixtures in a vacuum oven at 120 °C for 1 h, followed by further processing using compression molding, at 150–160 °C for 1–3 h (Figure S33). DSC isotherms performed on a mixture with a Zn(Oct)_2_/DBU ratio of 80:20 showed that the gel point was reached after 43 min at 120 °C (Figure S34a). When the temperature was increased to 150 °C, the moduli plateau was achieved (Figure S34b), with a *G*′ value of 1.59 MPa. This indicates a higher degree of transesterification and, consequently, a higher cross-linking density compared to the network obtained using only Zn(Oct)_2_ (i.e., *G*′ = 0.05 MPa). Notably, this improvement was achieved by incorporating just a small amount of DBU in the catalyst mixture. FTIR spectra of two dry, thick network films (Zn_60_/DBU_40_ and Zn_30_/DBU_70_) showed no significant structural differences and no evidence of unreacted COOH groups (Figure S35). The absence of a C=O stretch for COOH (1700–1730 cm^–1^) and the presence of a C=O stretch for ester (1755–1735 cm^–1^) confirm the complete reaction of COOH groups with DGEBA, within the detection limits of IR spectroscopy. In contrast, the COOH-functionalized polymer alone (P8-COOH) exhibited a shoulder in the C=O stretch peak (Figure S36), indicating the presence of both COOH and ester groups.

**Figure 4:**
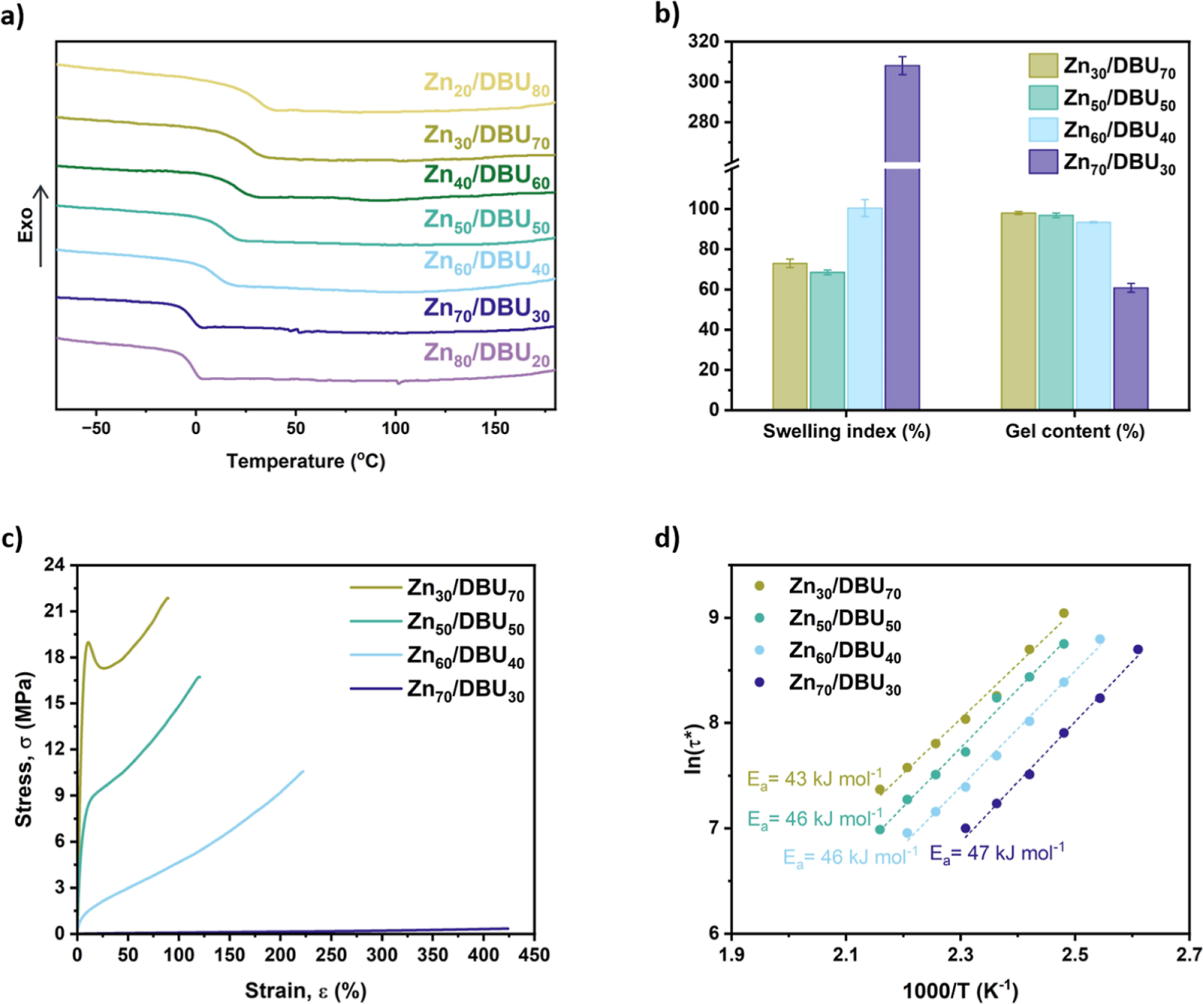
Characterization of the cross-linked networks obtained by varying the Zn(Oct)_2_/DBU catalyst ratio. (a) DSC curves of the first heating cycle of the sample after the curing process (exo up, normalized to the same heat flow per gram). (b) Swelling index and gel content calculated after swelling the polymer networks in THF for 24 h and drying for 96 h. (c) Representative stress–strain curves showing the tunability of the mechanical properties with the variation of the catalyst ratio (10 mm min^–1^ extension rate). (d) Plots of ln(τ*) as a function of 1000/*T* and linear fittings for the extrapolation of the activation energy of the dynamic bond exchange.

Based on the DSC studies, a shift in the *T*_g_ value toward higher temperatures was observed as the amount of DBU in the catalyst mixture increased, indicating an increase in the cross-linking density. By varying the Zn(Oct)_2_:DBU ratio, *T*_g_ values from 28 °C to −3 °C were obtained (Figure 4a and Table S7). Furthermore, all cross-linked materials exhibited a high thermal stability with the on-set of degradation occurring above 280 °C (Figure S37). Swelling experiments in THF were conducted on selected cured samples (Figure 4b). The data indicated that networks with more DBU (Zn_30_/DBU_70_, Zn_50_/DBU_50_ and Zn_60_/DBU_40_) had higher cross-linking densities, as evidenced by their high gel content (>90%) and the low overall swelling index (Table S8). Conversely, the network containing the most Zn(Oct)_2_, Zn_70_/DBU_30_, showed a gel content just above 60%, resulting in a significantly higher swelling (∼300%). These results confirmed previous findings observed using a single-catalyst system, where DBU proved to be a more effective catalyst for the transesterification reaction compared to Zn(Oct)_2_. A gel content higher than 90% was also observed in other solvents, such as ethanol or chloroform (Figure S38). This confirms that the network effectively retained its cross-linked structure, with all uncross-linked material being completely extracted.

This difference in network cross-linking also resulted in a range of mechanical properties, spanning from plastics, in the case of the sample containing the highest amount of DBU, to soft elastomers, for the material with the highest Zn content (Figure 4c and Table S9). In particular, increasing the DBU catalyst content led to a reduction in strain at break from 421 to 89%, while improving the ultimate tensile strength from 0.33 to 24.00 MPa, Young’s modulus from 0.26 to 418 MPa (determined from the gradient of stress–strain data), and toughness from 0.65 to 21.8 MPa.

Stress-relaxation experiments performed on the samples demonstrated relaxation times that decreased with increasing temperature (Figures S39–S42). By increasing the amount of Zn(Oct)_2_, the stress induced by deformation was more effectively released at elevated temperatures. This is attributed to a lower cross-linking density, resulting from a reduced degree of transesterification.

This confirms that higher Zn(Oct)_2_ content favors the formation of less rigid, more dynamic networks capable of better stress relaxation at higher temperatures. The activation energy was extrapolated, using the Arrhenius relationship, by plotting the ln(τ*) values as a function of 1000/*T* (Figure 4d). The *E*_a_ values ranged from 43 to 47 kJ mol^–1^, indicating that the catalyst ratio does not significantly impact the overall activation energy of the system. The mixed catalyst systems exhibited a lower *E*_a_ compared to the single-catalyst network, which had an *E*_a_ of 66 kJ mol^–1^. This suggests enhanced catalytic activity of Zn(Oct)_2_ in the presence of DBU, perhaps through coordination of the N-base at the Zn(II) site, which moderates its Lewis acidity.^59,60^*E*_a_ values in the same range have been reported for vinylogous urethanes,^61^ Schiff base covalent adaptable networks,^62^ and poly(diketoenamine) vitrimers.^63^ To confirm that associative dynamic bond exchange occurred, frequency sweep experiments were conducted from 100 to 180 °C (Figure S43). The results revealed a consistent *G*′ plateau across all temperatures, indicating constant cross-linking density in the materials and thereby confirming an associative transesterification mechanism.

### Dimensional Stability, Reproducibility and Reprocessability

**Figure 5:**
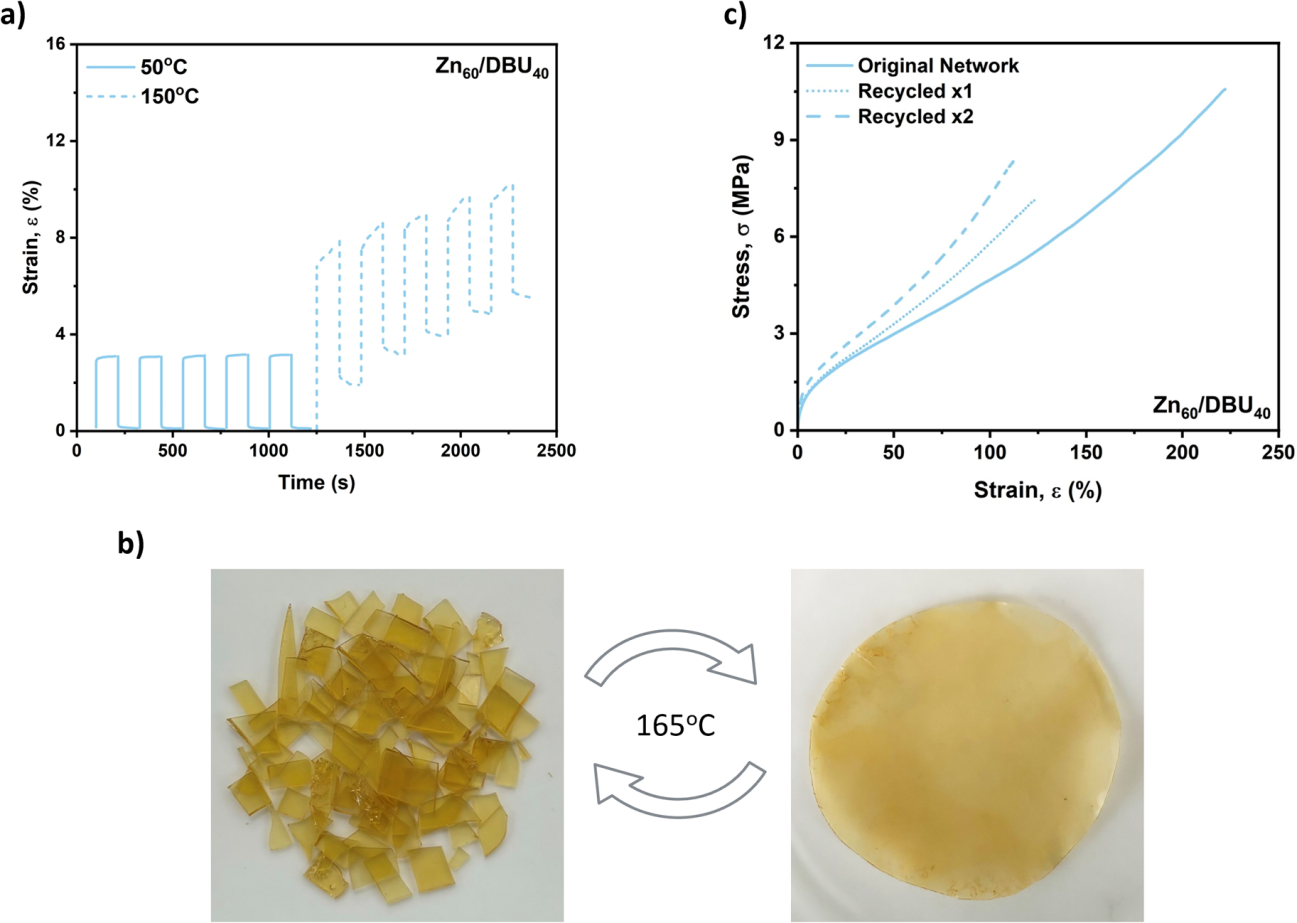
Determination of the dimensional stability and reprocessability of the polyester-based networks. (a) Cyclic creep recovery experiments performed on the Zn_60_/DBU_40_ sample at 50 °C (solid line) and 150 °C (dashed line). (b) Reprocessing by hot pressing at 165 °C for 1 h, starting from broken sample pieces. Only two recycling cycles were attempted. (c) Representative stress–strain curves of the Zn_60_/DBU_40_ network before and after two recycling cycles (10 mm min^–1^ extension rate).

The dimensional stability of the materials was determined by cyclic creep recovery experiments (Figures 5a and S44). The measurements were performed using a rheometer, where a stress of 0.01 MPa was applied for 100 s to each sample. Afterward, the stress was released for 100 s to allow the material to recover. This process was repeated for 5 cycles for each sample at both 50 and 150 °C. At 50 °C, all samples exhibited excellent creep recovery across all cycles (i.e., %recovery >90% in final cycle), where the extent of strain elongation under stress correlated with the content of cross-linking density. Increasing the temperature to 150 °C promoted dynamic bond exchange reactions leading to reduced creep recovery and diminished dimensional stability at the processing temperature. The reproducibility of the network was evaluated by synthesizing a second network with a Zn(Oct)_2_/DBU ratio of 60:40 and characterizing its thermomechanical properties. The resulting sample showed comparable properties to the original network, demonstrating the reliability of the method (Figure S45 and Table S10). Furthermore, the reprocessability of one of the polyester-based networks was assessed by breaking the sample into pieces, followed by compression molding the pieces, at 165 °C for 1 h (Figure 5b). The process was repeated for two cycles and the thermomechanical properties of the resulting materials were tested. The reprocessed materials showed slightly lower values for the strain at break, but higher Young’s modulus, suggesting that recycling induced further cross-linking within the network (Figure 5c and Table S11). To further investigate this, the twice reprocessed sample was analyzed by DSC and TGA. A slight increase in the *T*_g_ value, from 8 to 12 °C was observed, along with a decrease in the *T*_d,onset_ by approximately 20 °C (Figure S46). The rise in *T*_g_ was confirmed through DMA analysis, which also showed an increase in *E*′ in the rubbery plateau (*E'*_original_ = 4.80 MPa, and *E'*_recycledx2_ = 9.60 MPa), providing further evidence of the enhanced cross-linking density in the network after two recycling cycles (Figure S47 and Table S12). Stress-relaxation experiments revealed an increase in relaxation times, corresponding to a higher activation energy of 66 kJ mol^–1^ compared to 46 kJ mol^–1^ for the original network (Figure S48). This further confirms an increase in cross-linking density. No significant changes in the network structure were detected using FTIR spectroscopy, as the additional cross-linking from the transesterification reaction does not alter the overall network architecture (Figure S49). During the curing reaction, the extent of the cross-linking (i.e., transesterification reaction) decreases with conversion due to reduced network mobility. When the material is cut into pieces and then hot pressed, new areas come into contact, promoting additional reactions between newly exposed functionalities. This results in further cross-linking and, consequently, a material with reduced strain at break but greater stiffness. Overall, there was no significant difference in the properties of the two reprocessed samples indicating an overall good reprocessability of the polyester-based networks.

## Conclusions

Well-defined low molecular weight polyesters were synthesized via epoxide/anhydride ROCOP using commercial monomers without further purification. A polyester featuring 20% COOH-functionalization (i.e., 2 COOH per chain) was employed for the formation of polymer networks with the epoxy cross-linker DGEBA, and DBU or Zn(Oct)_2_ as the catalyst. DSC and rheological analyses demonstrated that DBU exhibited superior catalytic activity for the transesterification reaction, leading to the formation of a more highly cross-linked network with enhanced mechanical properties. In contrast, Zn(Oct)_2_ produced a less cross-linked network, which showed improved ability to dissipate applied stress as the temperature increased. These findings highlight the critical role of catalyst selection in controlling the thermomechanical properties and stress relaxation behavior of dynamic networks. Combining DBU and Zn(Oct)_2_ enabled the development of polymer networks with tunable thermomechanical properties, ranging from low-*T*_g_ soft elastomers, to higher-*T*_g_ plastics. The two-catalyst systems exhibited lower activation energies for the dynamic bond exchange compared to the single-catalyst network (43–47 vs 66 kJ mol^–1^, respectively). Furthermore, the materials showed excellent creep recovery at 50 °C, while the dynamic bond exchange was promoted at 150 °C. Finally, the reprocessability of the materials was demonstrated as a proof-of-concept. This work highlights the benefits of catalyst mixtures to better control cross-linking and transesterification reactions in the formation of vitrimers. Different combinations of catalysts can potentially be employed to enable the development of multifunctional materials and promote sustainability through selective recycling. Furthermore, the properties of the final networks may be tuned by incorporating other commercial epoxides or anhydrides, including biobased options, thereby helping improve their sustainability.

## Supporting Information Available

Full details on polymer and network synthesis and characterization; NMR, GPC, thermal, rheological and tensile data, and reaction methods (PDF)
