# Supplementary material for: Precise Carboxylic Acid-Functionalized Polyesters in Reprocessable Vitrimers
Source: J Am Chem Soc. 2025 Feb 14;147(8):6492–502. doi: 10.1021/jacs.4c14032 (PMC11869290; doi:10.1021/jacs.4c14032)
Supplement: Supplementary file 1 — ja4c14032_si_001.pdf [file ja4c14032_si_001.pdf]

Supporting Information

Precise Carboxylic Acid-Functionalized Polyesters in Reprocessable Vitrimers

Matilde Concilio[a], Gregory S. Sulley[a], Fernando Vidal[a], Steven Brown[b], Charlotte K. Williams[a]*

[a] Department of Chemistry, Chemistry Research Laboratory, University of Oxford, 12 Mansfield Road, Oxford OX1 3TA, UK

[b] Scott Bader Company Ltd, Wollaston, Wellingborough Northamptonshire, NN29 7RL, UK

Table of Contents

1. **Materials, Instruments, and General Methods** 4

Figure S1. Determination of the DSC sensitivity. (a) Schematic depiction of the reaction between benzyl glycidyl ether and 2-ethylhexanoic acid catalyzed by $Zn(Oct)_2$. 1,2-dimethoxybenzene was used to dilute the reaction mixture and decreased the overall concentration. (b) 1H NMR spectrum (400 MHz, $CDCl_3$) of the product obtained after running the d0 sample in the DSC, and determination of monomer conversion. (c) DSC curves of the d0-d3 sample dilutions (exo up, heating rate = 5 °C min^{-1})........ 6

Table S1. Epoxide concentration calculated for each dilution. 7

2. **Representative SA/epoxide(s) ROCOP** 8

Table S2. Reagents and quantities used in the synthesis of the copolymers of SA with vHO or vDO, and the co-epoxide BO. 9

3. **Representative COOH-functionalization via thiol-ene 'click' reaction** 9

4. **Representative synthesis of the polymer networks** 9

Table S3. Reagents, quantities, and curing conditions used in the synthesis of the polyester-based vitrimers. 9

5. **Synthesis and Characterization of SA/vHO Copolymers** 10

Scheme S1. Reaction scheme of the ROCOP of SA with vHO using different CTAs (diol, diacid, water); followed by COOH functionalization via thiol-ene reaction. (i) [cat]/[CTA]/[SA]/[vHO] = 1:16:200:300, in dry toluene, 100 °C, 24 h. (ii) COOH functionalization via thiol-ene reaction; [DMPA]/[alkene]/[thiol]= 0.2:1:2, dry THF, RT, UV, 1 h. 10

Figure S2. ^1H NMR spectrum (400 MHz, DMSO-d_6) of succinic anhydride showing the presence of 4.8 wt% of succinic acid impurities. 11

Figure S3. ^1H NMR spectrum (400 MHz, $CDCl_3$) of P1, obtained using the diol as CTA. 11

Figure S5. ^1H NMR spectrum (400 MHz, $CDCl_3$) of P3, obtained using the diacid as CTA and performed under air. 12

Figure S6. ^1H NMR spectrum (400 MHz, $CDCl_3$) of P4, obtained using the diacid and water as CTAs. 13

Figure S7. ^1H NMR spectrum (400 MHz, $CDCl_3$) of P5, obtained using water as CTA 13

Figure S8. SEC data (in THF) of (a) P1, (b) P2, (c) P3, (d) P4, and (e) P5, obtained using the diol, succinic acid or water as CTA, performed under argon or air 14

Figure S9. MALDI-TOF spectra and main polymer structures for P3 and P4 copolymers 15

Figure S10. (a) Plot of m/z vs N[th] repeat unit. MW_{theo} of the Nth repeat unit ($C_{10}H_{14}O_4$) = 198.21 g mol^{-1}; MW_{theo} of the end group ($C_{12}H_{22}O_3$) + K^+ = 253.42 g mol^{-1}. (b) Comparison of the experimental and theoretical isotope distributions for the peak at 2235 m/z, corresponding to the diacid-initiated 10th-mer having an additional ether linkage. 16

6. **Synthesis and Characterization of Low Viscosity Polyesters** 16

Figure S11. ^1H NMR spectrum (400 MHz, $CDCl_3$) of the vDO/SA copolymer P6. 16

Figure S12. ^1H NMR spectrum (400 MHz, $CDCl_3$) of the vHO/BO/SA copolymer P7. 16

Figure S13. ^{1}H NMR spectrum (400 MHz, $CDCl_3$) of the vDO/BO/SA copolymer P8 ... 17

Figure S14. Representative DOSY spectrum (500 MHz, $CDCl_3$) of the vDO/BO/SA copolymer P8. ... 17

Figure S15. ^{1}H NMR spectrum (400 MHz, $CDCl_3$) of P2-COOH ... 18

Figure S16. ^{1}H NMR spectrum (400 MHz, $CDCl_3$) of P6-COOH ... 18

Figure S17. ^{1}H NMR spectrum (400 MHz, $CDCl_3$) of P7-COOH ... 19

Figure S18. ^{1}H NMR spectrum (400 MHz, $CDCl_3$) of P8-COOH ... 19

Figure S19. SEC data (in THF) of the copolymers before (dashed line) and after COOH functionalization (solid line) for the copolymers obtained by copolymerization of SA with (a) VHO, (b) vDO, (c) vHO/BO, and (d) vDO/BO. 20

Figure S20. Thermogravimetric curves under air of the COOH-functionalized copolymers ... 20

Table S4. Degradation temperatures of the COOH-functionalized copolymers. ... 20

Figure S21. DSC traces of P2 (lilac) and P6 (teal) before (dashed line) and after (solid line) functionalization, showing a single thermal transition corresponding to a glass transition (exo up, normalized to the same heat flow per gram). 21

Figure S22 DSC traces of P7 (dark blue) and P8 (plum) before (dashed line) and after (solid line) functionalization, showing a single thermal transition corresponding to a glass transition (exo up, normalized to the same heat flow per gram). ... 21

Figure S23. Viscosity as a function of the shear rate measured at 30 °C, for (a) P2, (b) P6, (c) P7, and (d) P8 before (empty circle) and after functionalization (solid circle). The infinite viscosity values were obtained using the cross method. ... 21

7. **Synthesis and Characterization of Single-Catalyst Polyester Networks** ... 22

Figure S24. Image of the brittle films obtained using $P_{vDO100\%}$ and $P_{vDO50\%}$ polymers ... 22

Figure S25. Stress relaxation data for the crosslinked material obtained by reacting the hydroxyl-functionalized copolymer P8-OH (20% functionalization) with DGEBA, using $Zn(Oct)_2$ as the catalyst ([OH]/[epoxy]/[cat]= 1:1:0.05). ... 22

Figure S26. Study of the curing reaction as a function of temperature of mixtures of P8-COOH, DGEBA, and 5 mol% of $Zn(Oct)_2$ (dark blue), or DBU (light blue). (a) DSC data of the first heating cycle (exo up, normalized to the same heat flow per gram), and (b) oscillation temperature ramps. ... 22

Figure S27. Determination of the gelation point for the curing reaction of a mixture of P8-COOH, DGEBA and 5mol% of $Zn(Oct)_2$. DSC isotherms performed at (a) 120 °Cfor 1 h, and (b) 150 °Cfor 1 h ... 23

Figure S28. Determination of the gelation point for the curing reaction of a mixture of P8-COOH, DGEBA and 5mol% of DBU. DSC isotherm performed at 120 °Cfor 1 h ... 23

Figure S29. DMA temperature ramp profiles for the two polymer networks cured with (a) $Zn(Oct)_2$, and (b) DBU. ... 23

Table S5. Moduli crossover temperature and Tan(δ) peak temperature for the networks cured with $Zn(Oct)_2$ and DBU ... 24

Table S6. Summary of the mechanical properties of the P8-COOH/DGEBA cured materials obtained using $Zn(Oct)_2$ or DBU as catalyst. ... 24

Figure S30. Normalized stress relaxation data for the crosslinked material obtained by curing P8-COOH and DGEBA with DBU, highlighting the catalyst's limited effectiveness in promoting dynamic bond exchange. ... 24

8. **Synthesis and Characterization of Two-Catalyst Polyester Networks** ... 24

Figure S31. DSC curves of the first heating cycle of the exothermic curing processes of the reaction mixtures containing the 20%-functionalized P8-COOH copolymer, DGEBA, and 5 mol% of different $Zn(Oct)_2$/DBU ratios (exo up, normalized to the same heat flow per gram). ... 24

Figure S32. Oscillation temperature ramp of the reaction mixtures containing the 20%-functionalized P8-COOH copolymer, DGEBA, and different $Zn(Oct)_2$/DBU ratios. G' and G" were measured as a function of temperature and

the gelation temperature (T_{gel}) was determined at the moduli crossover point. T_{gel} was taken as the minimum temperature required for curing the polymer networks. 25

Figure S33. Determination of the curing conditions for the networks obtained by varying the $Zn(Oct)_2$/DBU ratio. DSC curves of the networks after the initial curing treatment in the oven at 120 °C for 1 h (solid line), and after the hot press treatment (dashed line). The disappearance of the exothermic peak in the dashed line indicates complete curing. Data are shown for P8-COOH/DGEBA mixtures with $Zn(Oct)_2$/DBU ratios of (a) 20:80, (b) 30:70, (c) 40:60, (d) 50:50, (e) 60:40, (f) 70:30, and (g) 80:20. 26

Figure S34. Determination of the gelation point for the curing reaction of a P8-COOH/DGEBA mixture containing $Zn(Oct)_2$:DBU= 80:20. DSC isotherms performed at (a) 120 °Cfor 1 h, and (b) 150 °Cfor 1 h. 27

Figure S35. FTIR analysis of dry films. (a) FTIR spectra of the Zn_{60}/DBU_{40} and Zn_{30}/DBU_{70} networks, and (b) enlarged view of the wavenumber region corresponding to the C=O stretch, highlighting only the peak associated with the ester C=O stretch. 27

Figure S36. FTIR analysis of dry films. (a) FTIR spectra of the Zn_{60}/DBU_{40} film, the COOH-containing polymer P8-COOH, and the epoxy crosslinker DGEBA. (b) enlarged view of the wavenumber region corresponding to the C=O stretch, highlighting the presence of a shoulder in the case of the polymer, corresponding to the COOH C=O stretch. 28

Table S7. Thermal properties of the crosslinked materials obtained by varying the $Zn(Oct)_2$/DBU ratio in the reaction mixture. 28

Figure S37. Thermogravimetric curves under air of the crosslinked materials obtained my curing P8-COOH and DGEBA with different $Zn(Oct)_2$/DBU ratios. 28

Table S8. Swelling experiments performed in THF for the crosslinked materials obtained my curing P8-COOH and DGEBA with different $Zn(Oct)_2$/DBU ratios. 29

Figure S38. Swelling index and gel content calculated after swelling the $Zn(Oct)_2$/DBU=30:70 polymer network in THF, chloroform, and ethanol for 24 h and drying for 96 h 29

Table S9. Mechanical properties of the crosslinked materials obtained by curing P8-COOH and DGEBA with different $Zn(Oct)_2$/DBU ratios. 29

9. **Study of the Dynamic Bond Exchange in the Two-Catalyst Polyester Networks** 30

Figure S39. Stress relaxation data for the crosslinked material containing $Zn(Oct)_2$/DBU= 30:70. (a) Normalized stress relaxation data, and (b) plot of $\ln(\tau^*)$ vs 1000/T with a linear fit to extract the activation energy. 30

Figure S40. Stress relaxation data for the crosslinked material containing $Zn(Oct)_2$/DBU= 50:50. (a) Normalized stress relaxation data, and (b) plot of $\ln(\tau^*)$ vs 1000/T with a linear fit to extract the activation energy. 30

Figure S41. Stress relaxation data for the crosslinked material containing $Zn(Oct)_2$/DBU= 60:40. (a) Normalized stress relaxation data, and (b) plot of $\ln(\tau^*)$ vs 1000/T with a linear fit to extract the activation energy. 30

Figure S42. Stress relaxation data for the crosslinked material containing $Zn(Oct)_2$/DBU= 70:30. (a) Normalized stress relaxation data, and (b) plot of $\ln(\tau^*)$ vs 1000/T with a linear fit to extract the activation energy. 31

Figure S43. Frequency sweep experiments performed between 100 °C and 180 °C for (a) Zn_{30}/DBU_{70}, (b) Zn_{50}/DBU_{50}, (c) Zn_{60}/DBU_{40}, and (d) Zn_{70}/DBU_{30}. 31

10. **Dimensional Stability, Reproducibility and Reprocessability of the Two-Catalyst Polyester Networks** 32

Figure S44. Cyclic creep recovery experiments performed at 50 °C (solid line) and 150 °C (dashed line) on the (a) Zn_{30}/DBU_{70}, (b) Zn_{50}/DBU_{50}, and (c) Zn_{70}/DBU_{30} networks. 32

Figure S45. Comparison of the thermomechanical properties between the two individual networks synthesized by curing P8-COOH, DGEBA and $Zn(Oct)_2$/DBU=60:40. (a) DSC data (exo up, normalized to the same heat flow per gram), and (b) representative stress–strain curves (10 mm min^{-1} extension rate). 32

Table S10. Mechanical properties of the two separate networks obtained by curing P8-COOH, DGEBA and $Zn(Oct)_2$/DBU=60:40. 32

Table S11. Mechanical properties of the Zn_{60}/DBU_{40} network before and after two recycling cycles. 33

Figure S46. Thermal properties of the Zn_{60}/DBU_{40} network before (solid line) and after the second reprocessing cycle (dashed line). (a) DSC data (exo up, normalized to the same heat flow per gram), and (b) thermogravimetric curves under air. 33

Figure S47. DMA temperature ramp profiles for (a) the original network, and (b) the network after two recycling cycles. 33

Table S12. Moduli crossover temperature and Tan(δ) peak temperature for the original and twice recycled networks. 33

Figure S48. Stress relaxation data for the crosslinked material containing $Zn(Oct)_2$/DBU= 60:40 after two recycling cycles. (a) Normalized stress relaxation data, and (b) plot of ln(τ*) vs 1000/T with a linear fit to extract the activation energy. 34

Figure S49. FTIR spectra of the Zn_{60}/DBU_{40} network before (solid line) and after the second reprocessing cycle (dotted line). 34

1. Materials, Instruments, and General Methods

Materials. Reagents were used as received, unless stated otherwise. Succinic anhydride (SA, > 99%), 1,2-epoxy-5-hexene (vHO, 97%), 1,2-epoxy-9-decene (vDO, 96%), 1,2-epoxybutane (BO, 99%), succinic acid (≥ 99%), the phosphazene base P1-tBu (≥ 97%), 3-mercaptopropionic acid (≥ 99%), diglycidyl ether of bisphenol A (DGEBA), and Amberchrom® 50WX8 (hydrogen form, 100-200 mesh) were purchased from Sigma Aldrich. Zinc bis(2-ethylhexanoate) ($Zn(Oct)_2$, Zn ~18%) was purchased from Fluorochem. 1,8-Diazabicyclo(5.4.0)undec-7-ene (DBU, ≥ 98%) was purchased from Thermo Fisher Scientific. 2,2-Dimethoxy-2-phenylacetophenone (DMPA, 99%, Sigma Aldrich) was recrystallized from toluene three times and stored under nitrogen. Solvents were dried through a solvent purification system, degassed by several freeze-pump-thaw cycles, and stored over 4 Å molecular sieves under nitrogen.

NMR Spectroscopy. NMR spectra were recorded at 25 °C on a Bruker Avance III HD nanobay 400 MHz spectrometer. The spectra were referenced internally to solvent signals. Chemical shifts are reported as parts per million relative to $SiMe_4$. Deuterated solvents (Cambridge Isotope Laboratories Inc.) were used as received.

Size exclusion chromatography (SEC). Polymer samples (2-10 mg) were dissolved in HPLC grade THF (1-1.2 mL). Before injection (100 μL), the samples were filtered through a PTFE membrane with 0.2 μm pore size. The samples were run on an Agilent 1260 Infinity II instrument equipped with two PLgel 5 μm MIXED-D 300 x 7.5 mm columns in series heated to 35 °C. The measurements were performed using THF as eluent at a flow rate of 1 mL min^{-1}. Samples were detected with a differential refractive index (RI) detector. Polystyrene standards (M_w 0.62-260.9 kg mol^{-1}) were used for the calibration. Agilent GPC/SEC software was used to analyze the data.

Differential Scanning Calorimetry (DSC). Purified polymer samples were analyzed on a DSC25 (TA Instruments) under a N_2 flow (50 mL min^{-1}). Samples were heated (10 °C min^{-1}) and equilibrated to 50 °C to remove their thermal history, then cooled to −80 °C (10 °C min^{-1}) before subjecting it to two further heating/cooling cycles from 50 to -80 °C at a rate of 10 °C min^{-1}. Glass transition temperatures (T_g) were determined from the midpoint of the transition in the third heating curve. The polymer networks were subjected to two heating/cooling cycles from 30 to 250°C at 5 °C min^{-1}.

The curing reactions were monitored by heating the reaction mixtures from 30 to 250°C at 5 °C min^{-1}. The DSC was calibrated using indium and zinc standards.

Determination of DSC sensitivity to study epoxy/COOH curing reaction. The sensitivity of DSC to monitor the curing reaction between the diacid-functionalized polymer and the diepoxy-containing crosslinker was assessed using small molecule models. Benzyl glycidyl ether (BGE) served as the model for the epoxy crosslinker, and 2-ethylhexanoic acid was used to represent the acid-containing polymer. $Zn(Oct)_2$ was employed as the catalyst (**Figure S1a**).

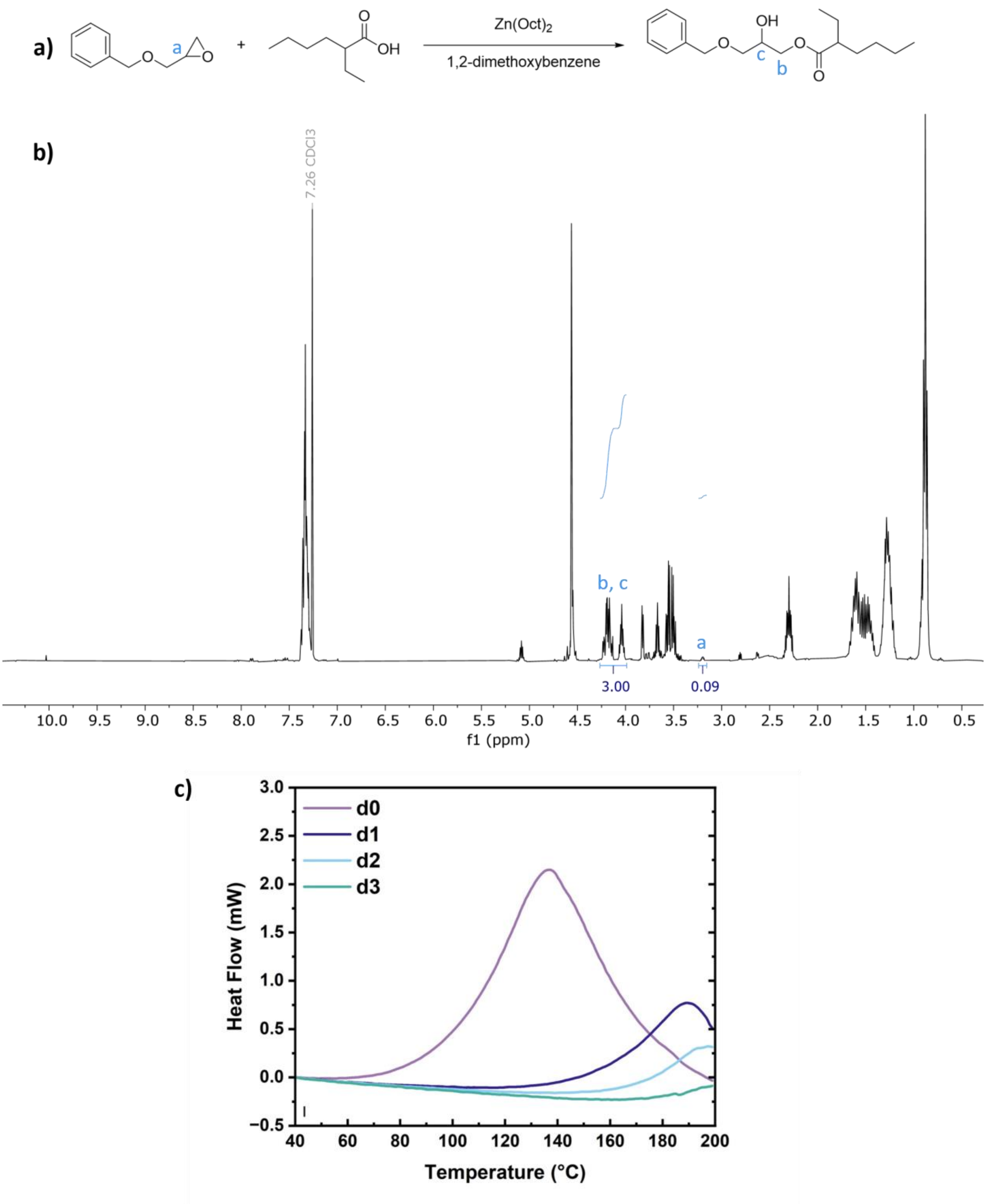

Figure S1. Determination of the DSC sensitivity. (a) Schematic depiction of the reaction between benzyl glycidyl ether and 2-ethylhexanoic acid catalyzed by $Zn(Oct)_2$. 1,2-dimethoxybenzene was used to dilute the reaction mixture and decreased the overall concentration. (b) 1H NMR spectrum (400 MHz, $CDCl_3$) of the product obtained after running the d0 sample in the DSC, and determination of monomer conversion. (c) DSC curves of the d0-d3 sample dilutions (exo up, heating rate = 5 °C min^{-1}).

The [COOH]:[epoxy]:[cat] ratio was maintained at 1:1:0.05. Reaction mixtures with varying concentrations were prepared by diluting with 1,2-dimethoxybenzene, a high boiling point solvent. The epoxide concentration in each dilution was calculated and summarized in **Table S1**, with the epoxide concentration in the polymer networks determined to be $1.27{\cdot}10^{-3}$ mol g^{-1}.

Table S1. Epoxide concentration calculated for each dilution.

Sample	Epoxide concentration [mol g^{-1}]
d0	$3.07 \cdot 10^{-3}$
d1	$1.55 \cdot 10^{-3}$
d2	$1.00 \cdot 10^{-3}$
d3	$5.65 \cdot 10^{-4}$
Polymer Networks	$1.27 \cdot 10^{-3}$

DSC measurements were conducted for each dilution by heating the reaction mixture from 30 to 200°C at 5 °C min^{-1}. Due to the solvent's boiling point of 207 °C, the maximum achievable temperature was 200 °C. The d0 sample, being the only one neat, was analyzed using ^1H NMR spectroscopy to determine the monomer conversion (**Figure S1b**). A COOH/epoxy conversion of 92% was calculated by integrating the peak at 3.99-4.27 ppm, corresponding to the three highlighted protons of the product, and the peak at 3.20 ppm, corresponding to the single proton of the unreacted epoxide. This result suggests that with a 34-min temperature ramp achieving 92% conversion, the curing process used for the synthesis of the polyester-based vitrimers should lead to a nearly complete conversion.

Furthermore, the detection limit of the DSC instrument is 0.1 mW, and all curves exhibit signals above this threshold (**Figure S1c**). Since the epoxide concentration in the polymer networks falls within the high concentration range, between d0 and d1, where detectable signals are still produced, the curing reaction is certainly detectable using DSC.

Thermogravimetric Analysis (TGA). Thermogravimetric analyses were performed on a TGA5500 System (TA Instruments), equipped with the TRIOS software package. The polymer samples (5-15 mg) were heated from 30 to 800 °C at a rate of 10 °C min^{-1} under air (25 mL min^{-1}).

MALDI-TOF Mass Spectrometry. MALDI-TOF analyses were conducted on a Bruker MALDI-MS Autoflex set to positive ion reflectron mode. Samples solutions of polymer (10 mg mL^{-1} in THF), dithranol (10 mg mL^{-1} in THF), and KTFA (10 mg mL^{-1} in THF) were premixed in a 1:4:1 ratio. Each sample was spotted three times onto a metal plate and allowed to dry completely before analysis.

Rheological analyses. The rheological analyses were conducted on a TA ARES-G2 instrument using polymer samples between 8- or 25-mm stainless steel plates. Flow sweep experiments were performed at 30 °C over a shear rate range from 10^{-4} to 10^{3} s^{-1}. The infinite viscosity values were obtained by using the cross method. The temperature sweep experiments on the curing processes were conducted using 20-mm disposable aluminum plates. The samples were heated from 30 to 250 °C, at a constant heating rate of 2 °C min^{-1}. The experiment was conducted in oscillatory mode with a strain amplitude of 1% and a frequency of 1 Hz. Oscillatory time sweep experiments were performed with 8-mm plates at 120 °C and 150 °C for 1h, using a strain amplitude of 1% and a frequency of 1 Hz. Stress relaxation experiments at different temperatures (100-200 °C, with intervals of +10°C) were performed using an 8-mm plate-plate geometry. After a 20 min temperature equilibration, a 2% strain was applied and the relaxation modulus (G(t)) was monitored over time. For frequency sweep measurements at different temperatures (100-180 °C, with intervals of +20°C), a strain of 1% with a normal force of 1 N

was applied and a frequency range from 10^{-2} to 10^{2} rad·s^{-1} was screened by following the evolution of G′ and G″ at a constant temperature. Cyclic creep experiments at different temperatures (50 and 150 °C) were performed using a constant force of 1 N. A shear stress of 0.01 MPa was applied to the sample for 100 seconds. After this period, the stress was removed, and the material was allowed to recover for an additional 100 seconds. This cycle was repeated five times for each sample. Prior to each rheological experiment, strain sweep experiments were performed to ensure that the applied stress was in the linear viscoelastic region at the measured temperatures.

Dynamic Mechanical Analysis (DMA). Thermal analysis (DMTA) was carried out using a DMA850 (TA Instruments), using an ACS III cooling system. Specimens of uniform width (5.3 mm) were cut using two parallel blades. Samples were heated from -80 °C to 200 °C, at a rate of 3 °C min^{-1}, with a frequency of 1 Hz, 0.1 N pre-load force, and 0.1% strain amplitude.

Tensile tests. The mechanical properties of the polymer networks were determined using a 68TM-10 model, 6800 series universal testing instrument (Instron). Dumbbell-shaped specimens were cut using a Zwick ZCP020 cutting press equipped with a cutting device for ISO 527-2 type 5B. Uniaxial extension experiments (10 mm min^{-1} cross-head speed) were run according to ISO 527. Five technical replicates were performed per sample.

Swelling tests. Samples of 8 mm diameter and 1-0.5 mm thickness, with a weight of 20 to 40 mg, were dissolved in THF, chloroform or ethanol (2.5 mL). The tests were carried out for 24 h, at room temperature. Subsequently, the solvent was removed, and the samples were dried under vacuum, for 96 h at 40 °C. Three technical replicates were performed per sample. The swelling index was calculated using equation (1), and the gel content was calculated using equation (2).

$$\text{Swelling Index (\%)} = [(w_s - w_i)/w_i] \times 100 \quad (1)$$

$$\text{Gel Content (\%)} = (w_d/w_i) \times 100 \quad (2)$$

with w_i= initial weight of sample, w_s= weight of swollen sample, w_d= weight of dried sample.

Reprocessability tests. The network was reprocessed by hot pressing. The sample was first cut into small pieces and placed between two metal plates sandwiched with Teflon sheets. The material was then pressed at 165 °C, for 1 h, under 1 ton m^{-2} and allowed to cool overnight.

Fourier Transform Infrared (FTIR) Spectroscopy. FTIR spectra were recorded of dry, thick polymer films on a Bruker Tensor 27 spectrophotometer, using 32 scans from 4000 to 600 cm^{-1}, at a resolution of 4 cm^{-1}.

2. Representative SA/epoxide(s) ROCOP

Inside a nitrogen filled glovebox, the catalyst P1-tBu (284 µL, 1.24 mmol, 0.5 eq) and anhydrous toluene (43 mL) were transferred into an oven-dried vial, which was sealed and moved into a fume hood. The catalyst solution was then transferred in an oven-dried round bottom flask equipped with a magnetic stirrer, containing SA (24.8 g, 247 mmol, 100 eq), BO (25.8 mL, 297 mmol, 120 eq), and vDO (13.6 mL, 74 mmol, 30 eq). The final monomer concentration was 7.5 M. The concentration of diacid was adjusted considering the amount of succinic acid already present in the specific

batch of anhydride used. In this case, SA contained 2 wt% of diacid (determined by ^{1}H NMR spectroscopy), therefore, an additional 1.83 g (15.5 mmol, 6.3 eq) of succinic acid were added to achieve a final DP of 10. The reaction mixture was deoxygenated, with an argon flow for 20 min, before being heated to 100 °C for 24 h. The polymerization was quenched by exposing the reaction mixture to air and cooling it to room temperature. The polymer was purified by adding Amberchrom® 50WX8 (~ 10 g) and stirring overnight at room temperature. The solid was filtered and the solution passed through a silica plug before removing the solvent in vacuo. ^{1}H NMR spectra of the crude and purified products were measured in $CDCl_3$. SEC samples were prepared by dissolving the purified product in HPLC grade THF and filtered before use.

Table S2. Reagents and quantities used in the synthesis of the copolymers of SA with vHO or vDO, and the co-epoxide BO.

Sample	P1-tBu (μL/ mmol)	Diacid added (g/mmol)	SA (g/mmol)	vHO (mL/ mmol)	vDO (mL/ mmol)	BO (mL/ mmol)	Toluene (mL)
**P1**[a]	8/0.04	-	0.72/7	1.2/11	-	-	1.2
**P2**	33/ 0.14	0.13/1.1	2.9/29	4.8/43	-	-	4.7
**P3**	9/ 0.04	0.032/0.3	0.80/8	1.3/11	-	-	1.2
**P4**[b]	9/0.04	-	0.80/8	1.3/11			1.2
**P5**[b]	9/0.04	-	0.80/8	1.3/11			1.2
**P6**	9/0.04	0.032/0.3	0.76/8	-	2.1/11	-	0.5
**P7**	110/0.5	0.85/7.2	9.91/99	3.4/30	-	10.3/119	19.3
**P8**	135/0.6	0.87/7.4	11.76/118	-	6.5/35	12.3/141	20.4
**$P_{vDO100\%}$**	56/0.2	0.36/3.1	4.90/49	-	13.5/73	-	2.8
**$P_{vDO50\%}$**	158/0.7	1.02/8.7	13.82/138	-	19.0/104	9.0/104	18

[a] 1,4-benzenedimethanol (BDM) was used as CTA (0.04 g/0.3 mmol). [b] Water was used as CTA (5 μL/0.3 mmol for P4; 10 μL/ 0.6 mmol for P5).

3. Representative COOH-functionalization via thiol-ene 'click' reaction

Inside a nitrogen filled glovebox, 2,2-dimethoxy-2-phenylacetophenone (1.17 g, 5.6 mmol, 0.2 eq) was dissolved in anhydrous THF (33 mL) into an oven-dried vial, which was sealed and moved into a fume hood. The photoinitiator mixture was then transferred into an oven-dried round bottom flask equipped with a magnetic stirrer, containing the P8 polymer (33.3 g) and 3-mercaptopropionic acid (5.9 mL, 45.5 mmol, 2 eq). The solution was then diluted to a final polymer concentration of 100 mg mL^{-1} by adding dry THF (300 mL). Subsequently, the reaction mixture was deoxygenated, with an argon flow for 20 min, and the thiol-ene reaction was induced by UV irradiation (365 nm, 10W, at 25 °C, for 1h). The final polymer was purified by repetitive precipitation into a 2:1 diethyl ether:hexane mixture.

4. Representative synthesis of the polymer networks

The COOH-functionalized copolymer P8-COOH (1.80 g, 8.51 mmol, 1 eq of COOH groups), $Zn(Oct)_2$ (0.15 g, 0.43 mmol, 0.05 eq with respect to COOH groups), and DGEBA (1.45 g, 4.25 mmol, 1 eq of epoxy groups) were first mixed in a glass vial at 80 °C. The homogeneous mixture was then poured in a Teflon mold and partially cured in a vacuum oven at 120 °C for 1 h. Subsequently, the crosslinking reaction was completed in a hot press at 170 °C for 1 h. The resulting film was slowly cooled to room temperature.

Table S3. Reagents, quantities, and curing conditions used in the synthesis of the polyester-based vitrimers.

Entry	$Zn(Oct)_2$:DBU*	P8-COOH (g)	DGEBA (g)	$Zn(Oct)_2$ (mg)	DBU (μL)	Curing conditions

**1**	100:0	1.45	1.16	120	-	170 °C, 1h
**2**	0:100	1.98	1.59	-	70	150 °C, 1h
**3**	20:80	1.82	1.47	30	52	150 °C, 1h
**4**	30:70	2.06	1.69	52	52	150 °C, 1h 45min
**5**	40:60	2.18	1.79	74	47	150 °C, 1h 45min
**6**	50:50	1.93	1.55	80	34	150 °C, 2h
**7**	60:40	2.07	1.70	105	30	150 °C, 2h
**8**	70:30	2.09	1.71	123	23	150 °C, 3h
**9**	80:20	1.97	1.58	131	14	160 °C, 1h

* The overall [cat]:[COOH] ratio was kept constant to 0.05:1.

5. Synthesis and Characterization of SA/vHO Copolymers

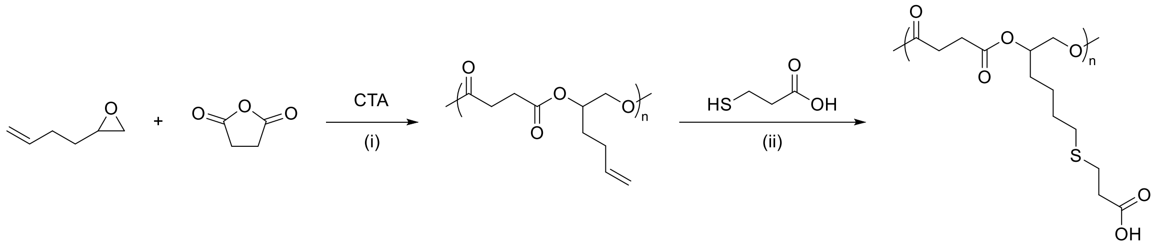

Scheme S1. Reaction scheme of the ROCOP of SA with vHO using different CTAs (diol, diacid, water); followed by COOH functionalization via thiol-ene reaction. (i) [cat]/[CTA]/[SA]/[vHO] = 1:16:200:300, in dry toluene, 100 °C, 24 h. (ii) COOH functionalization via thiol-ene reaction; [DMPA]/[alkene]/[thiol]= 0.2:1:2, dry THF, RT, UV, 1 h.

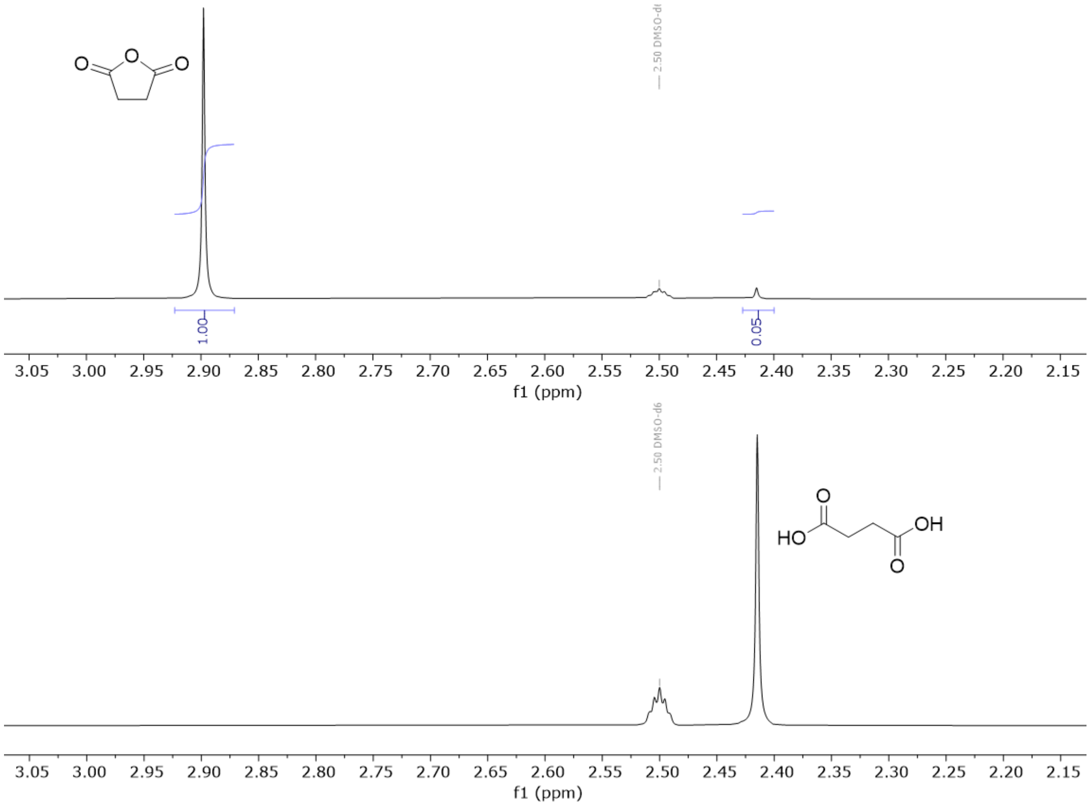

Figure S2. ^{1}H NMR spectrum (400 MHz, DMSO-d$_6$) of succinic anhydride showing the presence of 4.8 wt% of succinic acid impurities.

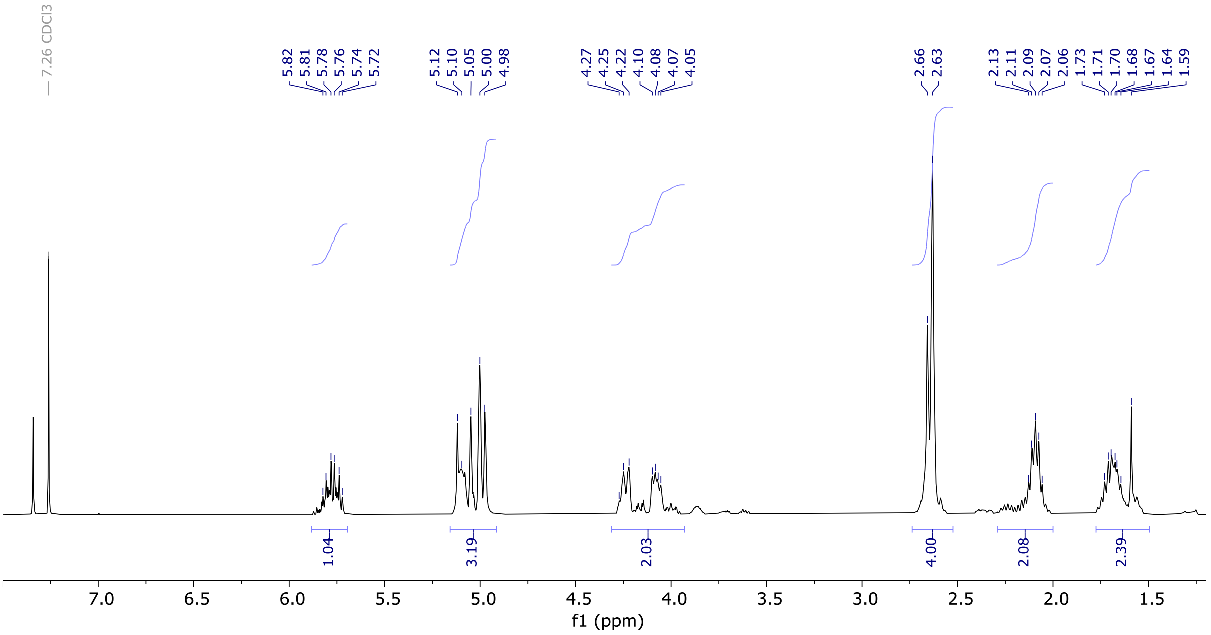

Figure S3. ^{1}H NMR spectrum (400 MHz, $CDCl_3$) of P1, obtained using the diol as CTA.

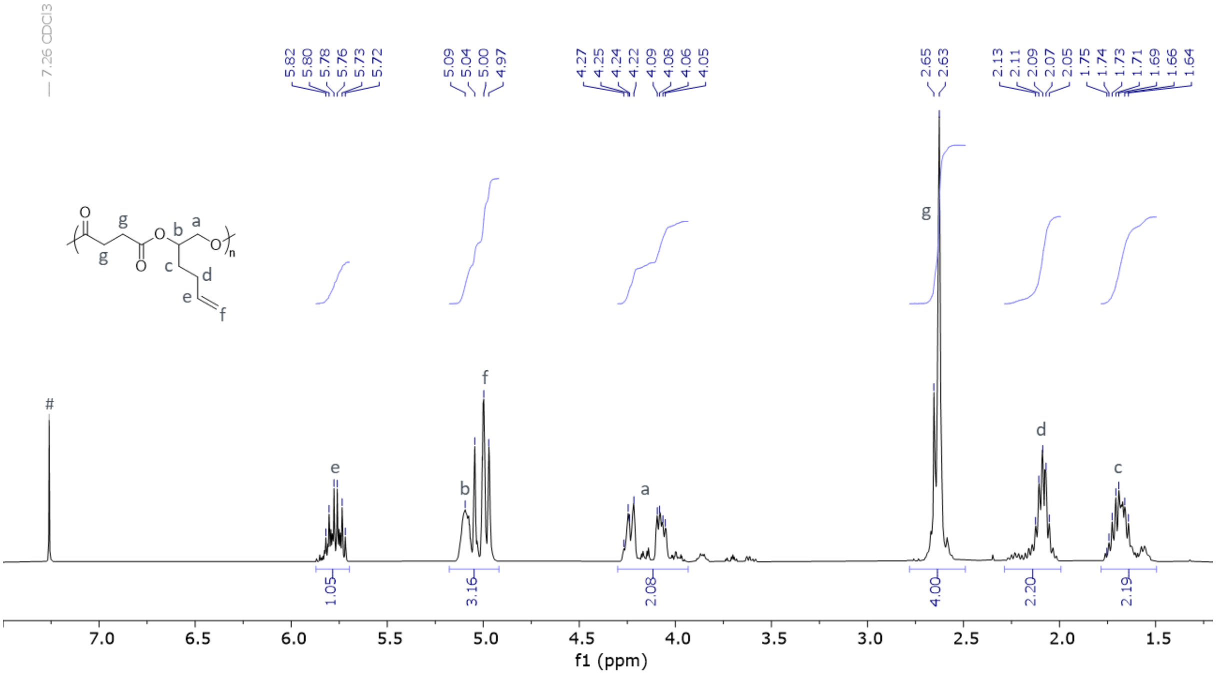

Figure S4. ^{1}H NMR spectrum (400 MHz, $CDCl_3$) of P2, obtained using the diacid as CTA.

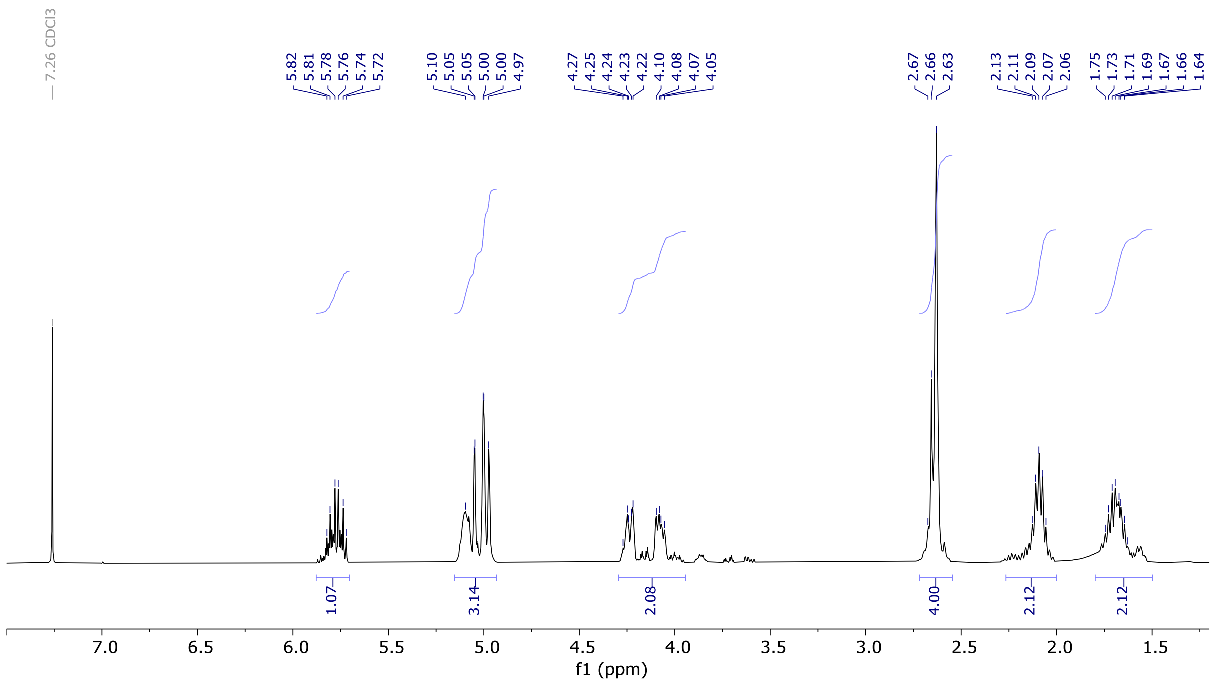

Figure S5. ^{1}H NMR spectrum (400 MHz, $CDCl_3$) of P3, obtained using the diacid as CTA and performed under air.

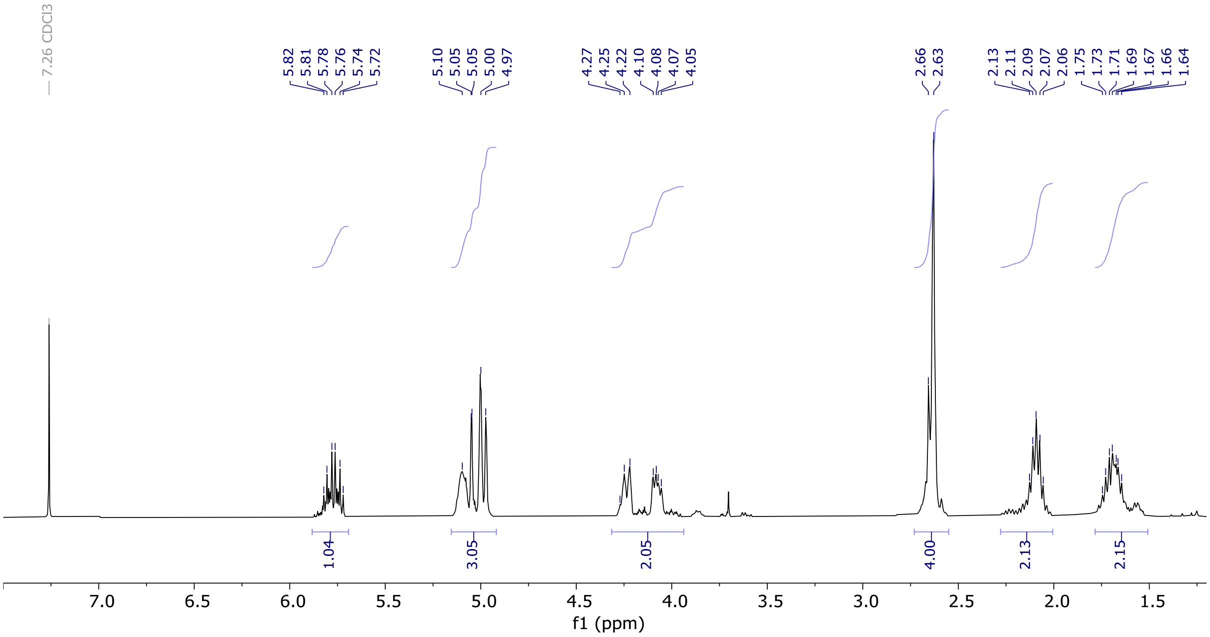

Figure S6. ^{1}H NMR spectrum (400 MHz, $CDCl_3$) of P4, obtained using the diacid and water as CTAs.

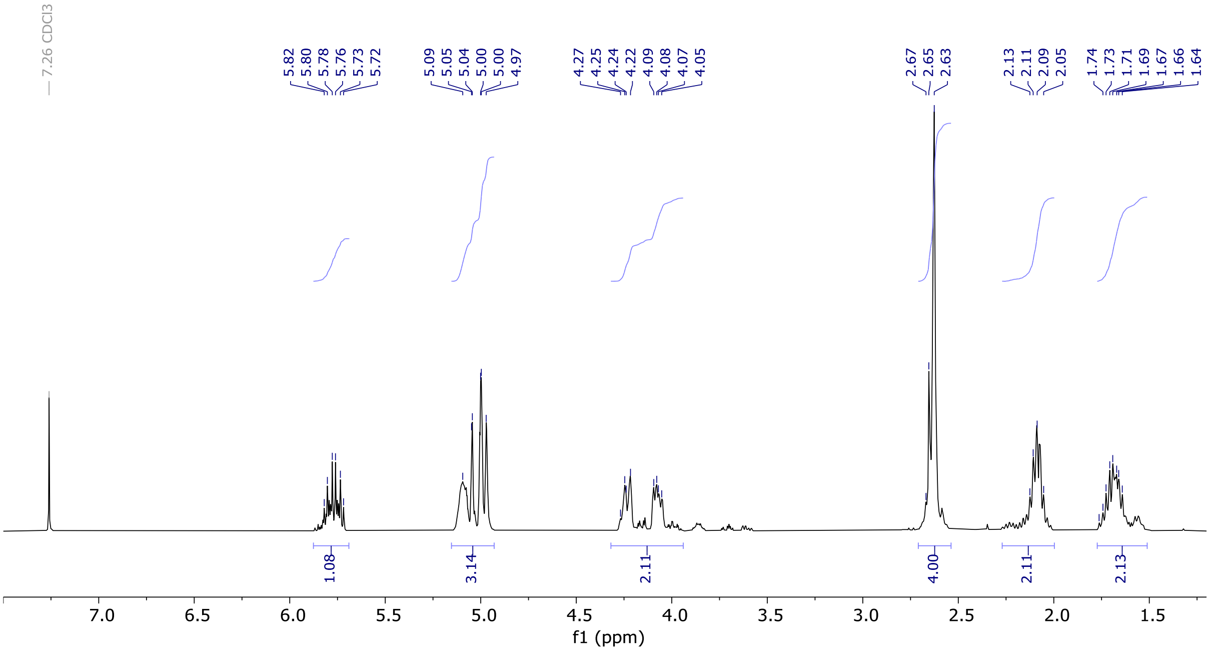

Figure S7. ^{1}H NMR spectrum (400 MHz, $CDCl_3$) of P5, obtained using water as CTA.

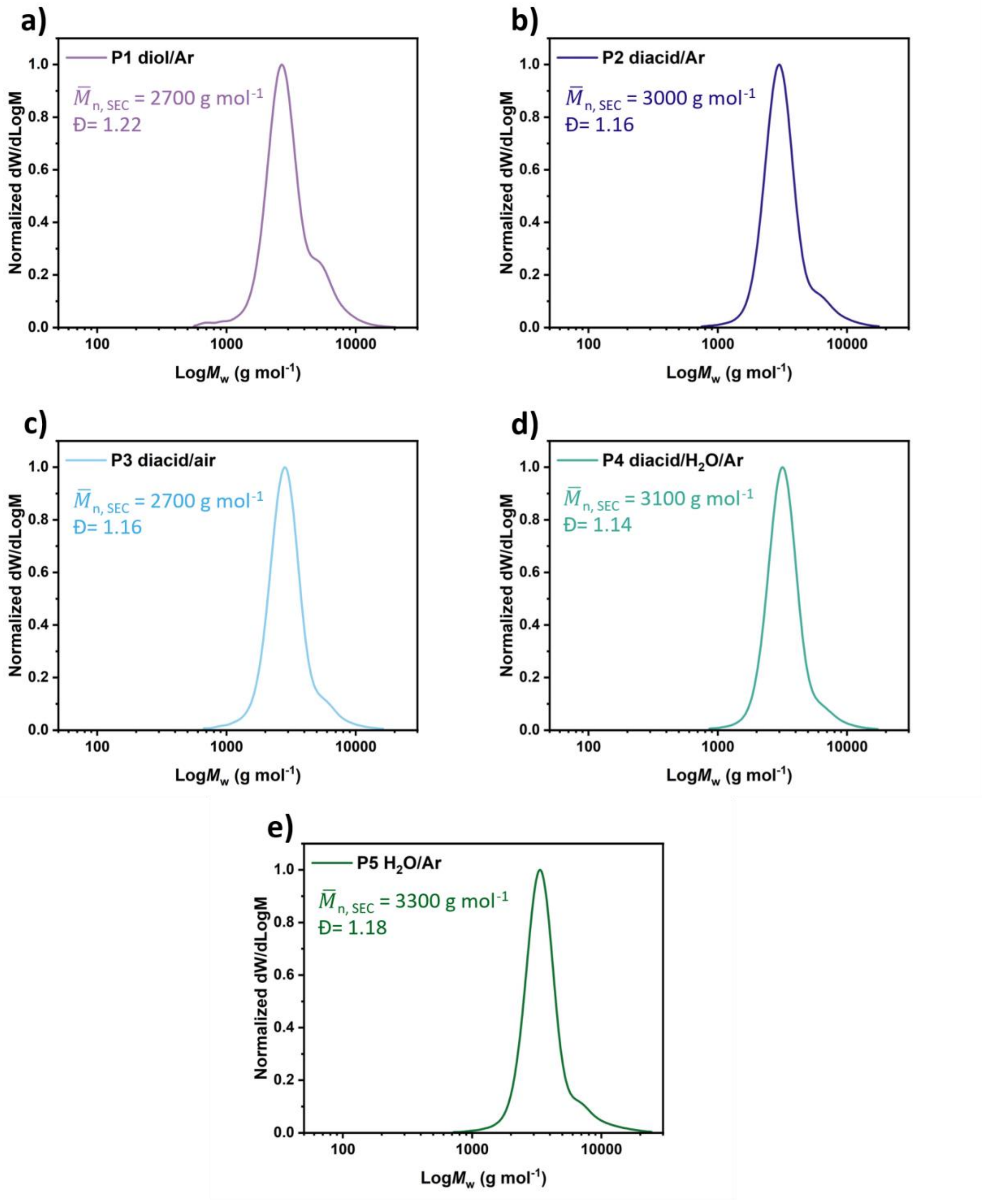

Figure S8. SEC data (in THF) of (a) P1, (b) P2, (c) P3, (d) P4, and (e) P5, obtained using the diol, succinic acid or water as CTA, performed under argon or air.

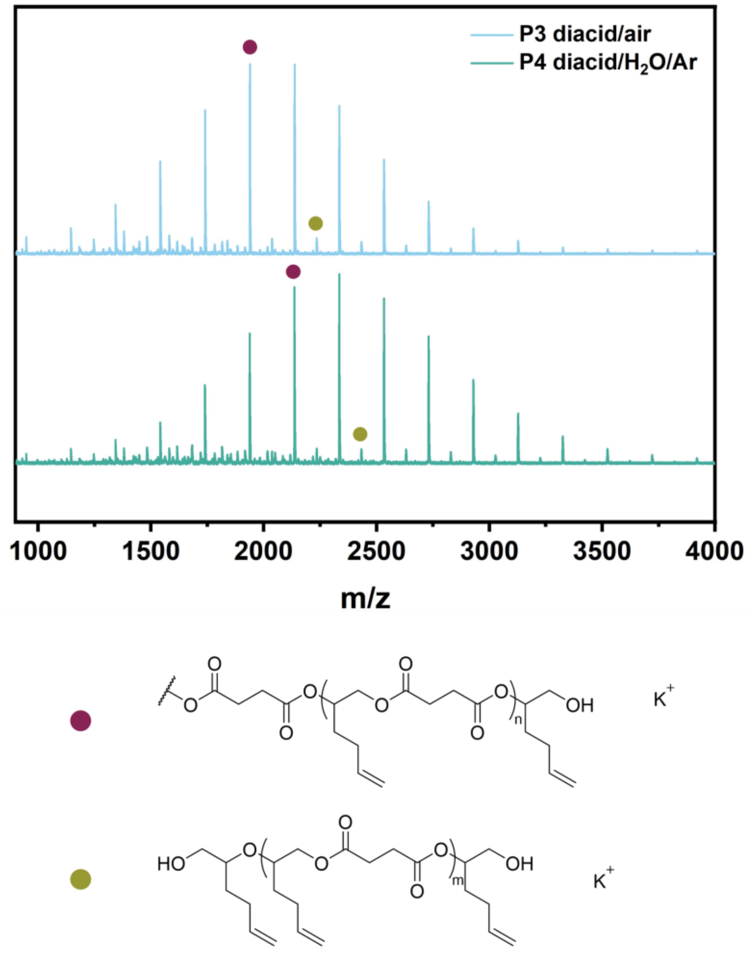

Figure S9. MALDI-TOF spectra and main polymer structures for P3 and P4 copolymers.

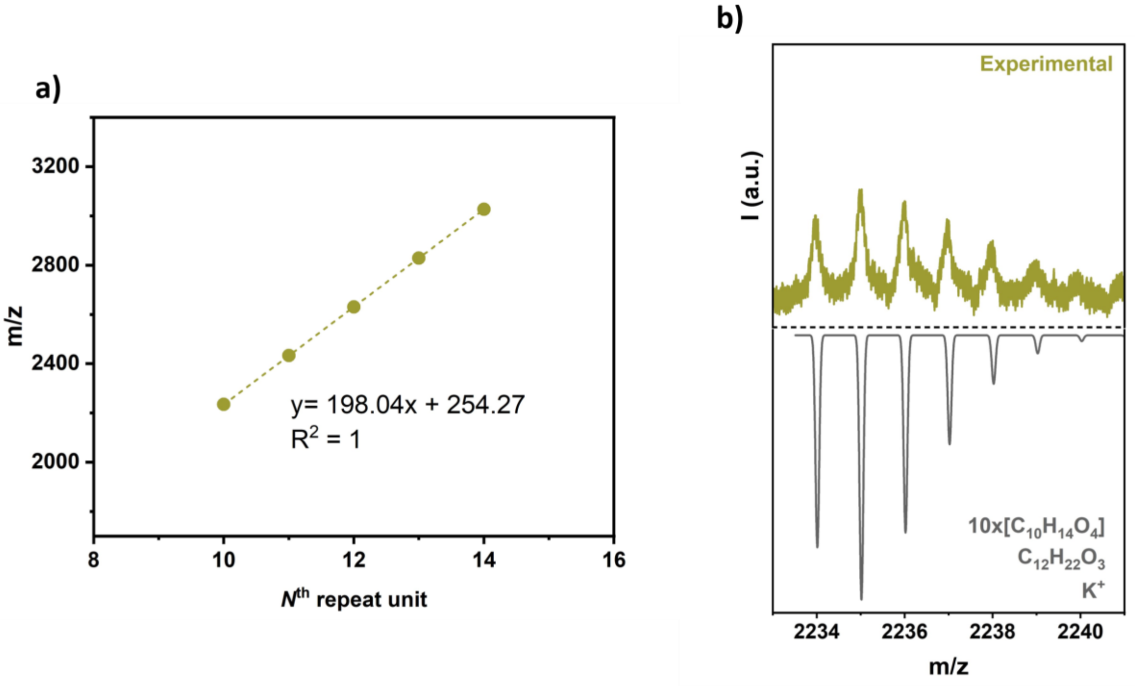

Figure S10. (a) Plot of m/z vs N[th] repeat unit. MW_{theo} of the Nth repeat unit ($C_{10}H_{14}O_4$) = 198.21 g mol^{-1}; MW_{theo} of the end group ($C_{12}H_{22}O_3$) + K^+ = 253.42 g mol^{-1}. (b) Comparison of the experimental and theoretical isotope distributions for the peak at 2235 m/z, corresponding to the diacid-initiated 10th-mer having an additional ether linkage.

6. Synthesis and Characterization of Low Viscosity Polyesters

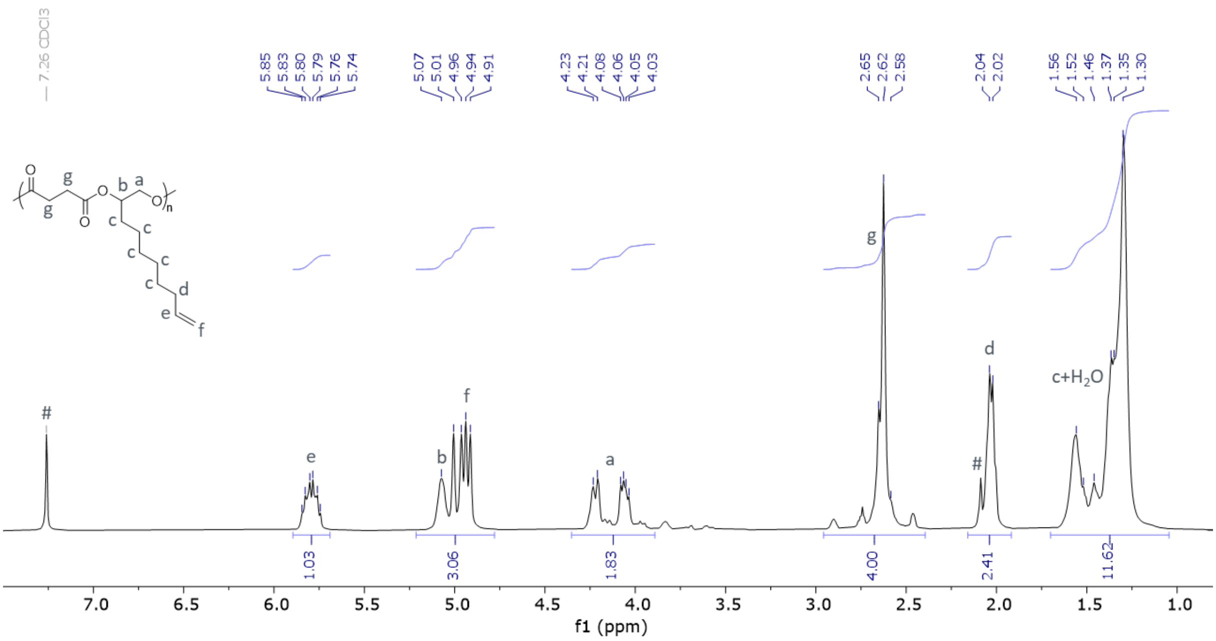

Figure S11. ^1H NMR spectrum (400 MHz, $CDCl_3$) of the vDO/SA copolymer P6.

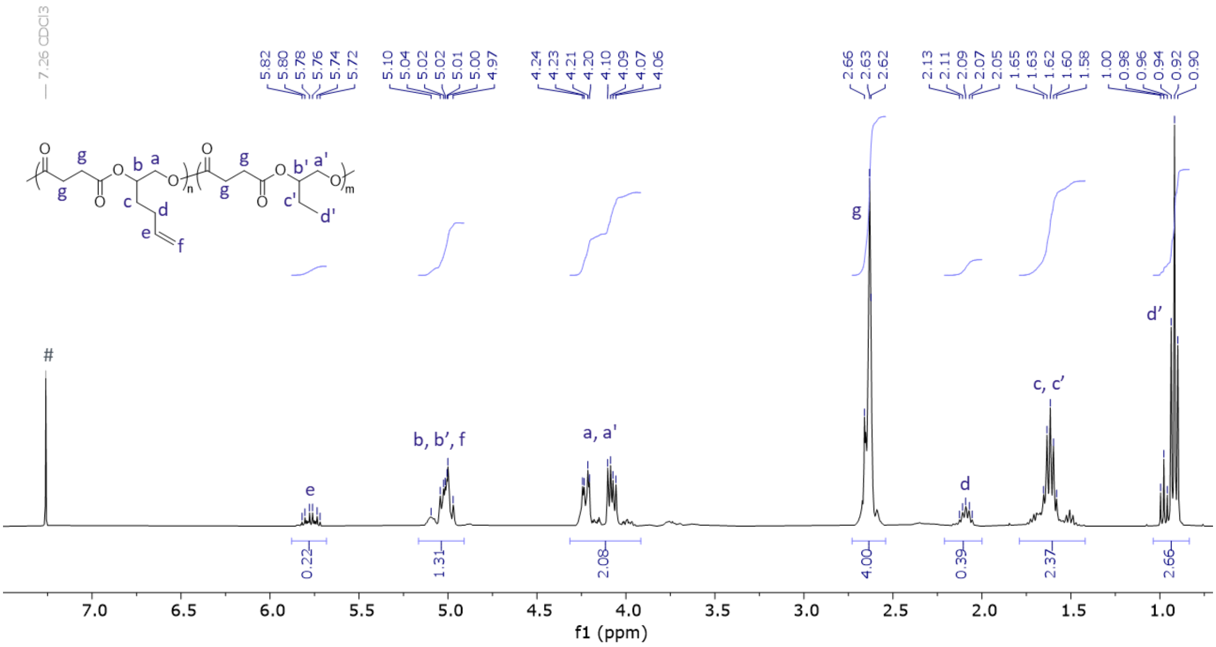

Figure S12. ^1H NMR spectrum (400 MHz, $CDCl_3$) of the vHO/BO/SA copolymer P7.

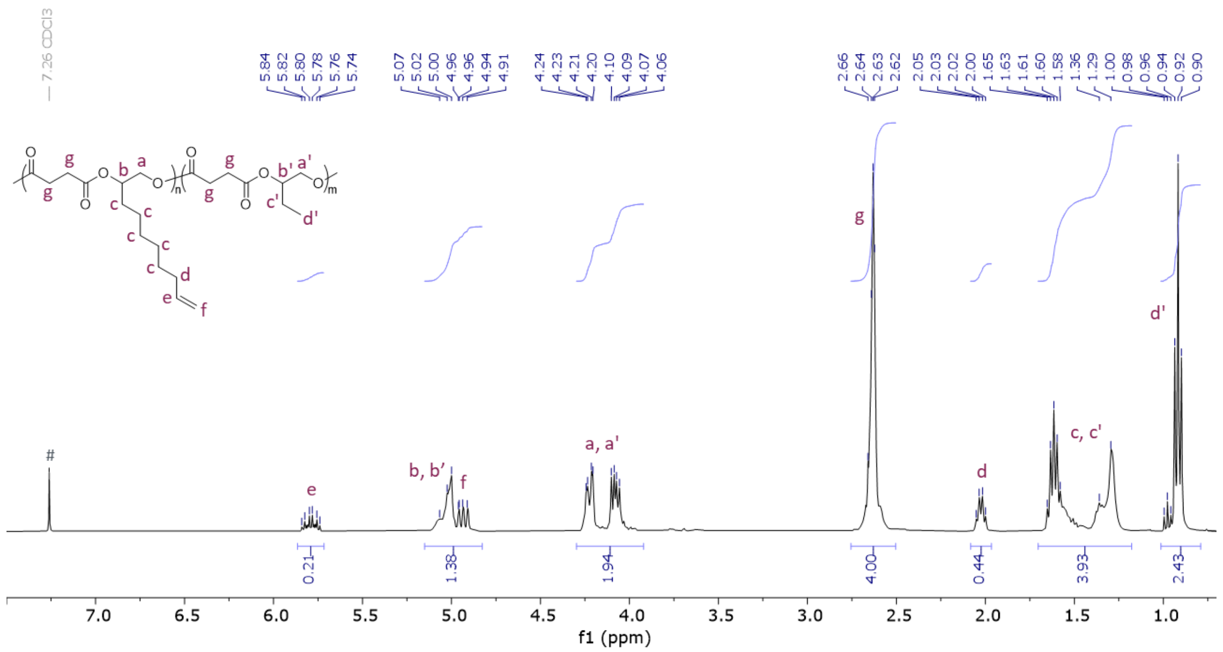

Figure S13. ^{1}H NMR spectrum (400 MHz, $CDCl_3$) of the vDO/BO/SA copolymer P8.

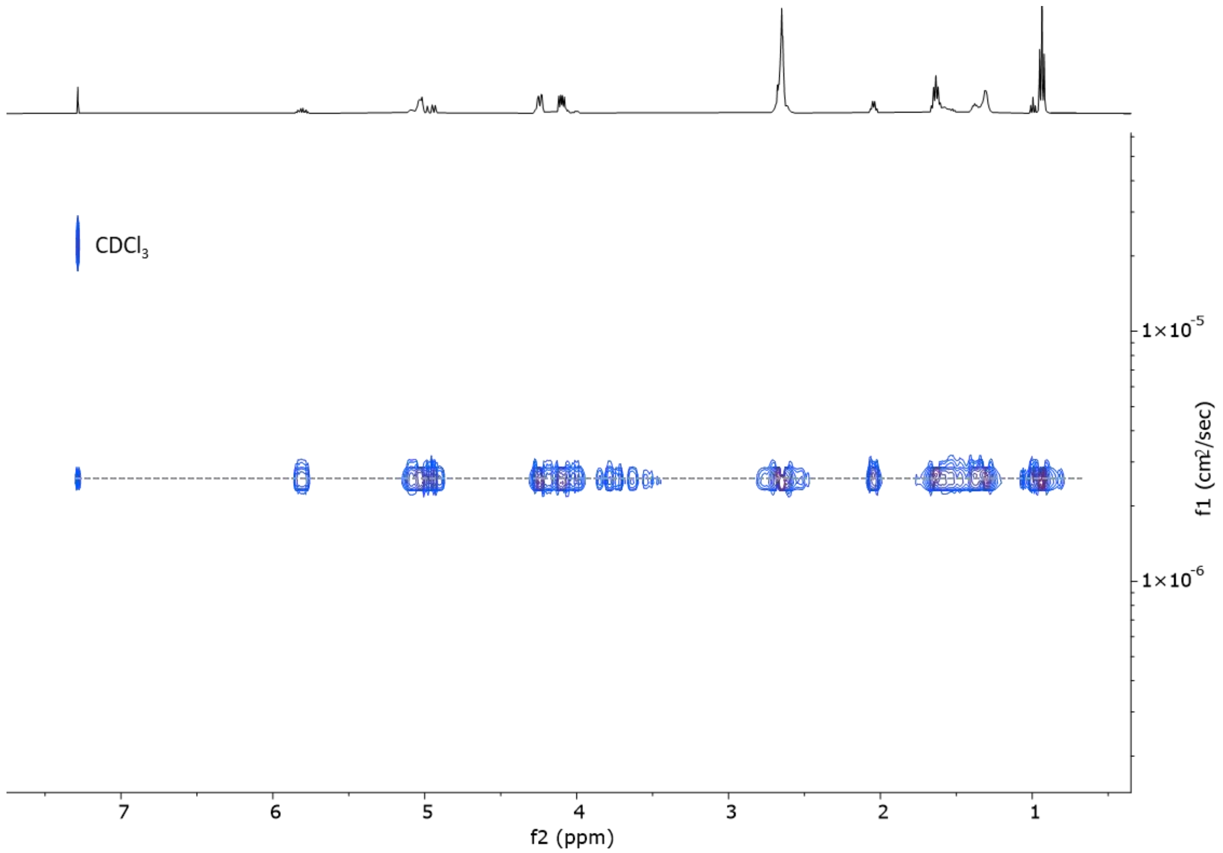

Figure S14. Representative DOSY spectrum (500 MHz, $CDCl_3$) of the vDO/BO/SA copolymer P8.

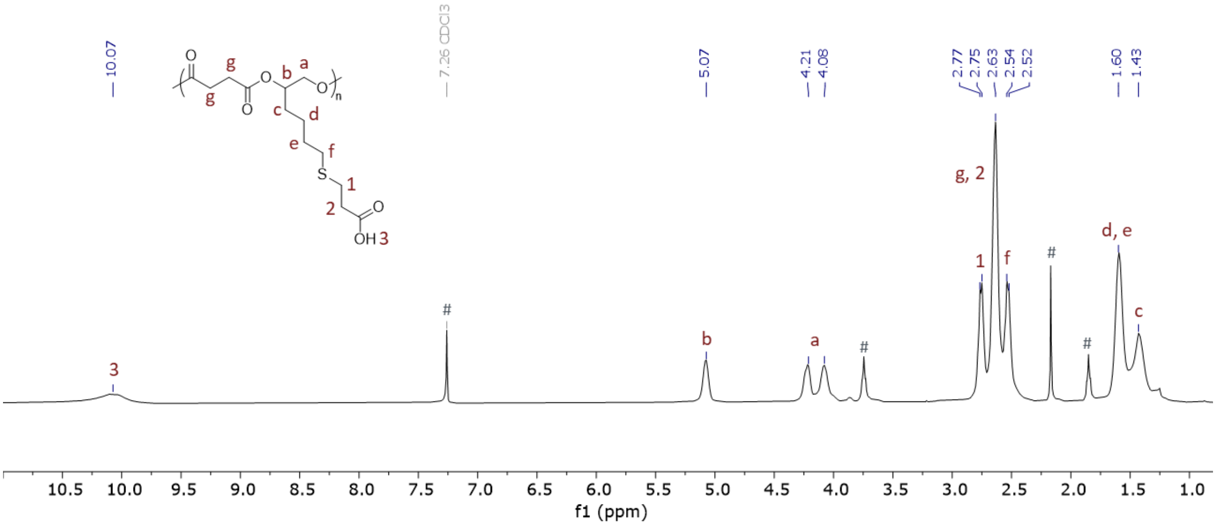

Figure S15. ^{1}H NMR spectrum (400 MHz, $CDCl_3$) of P2-COOH.

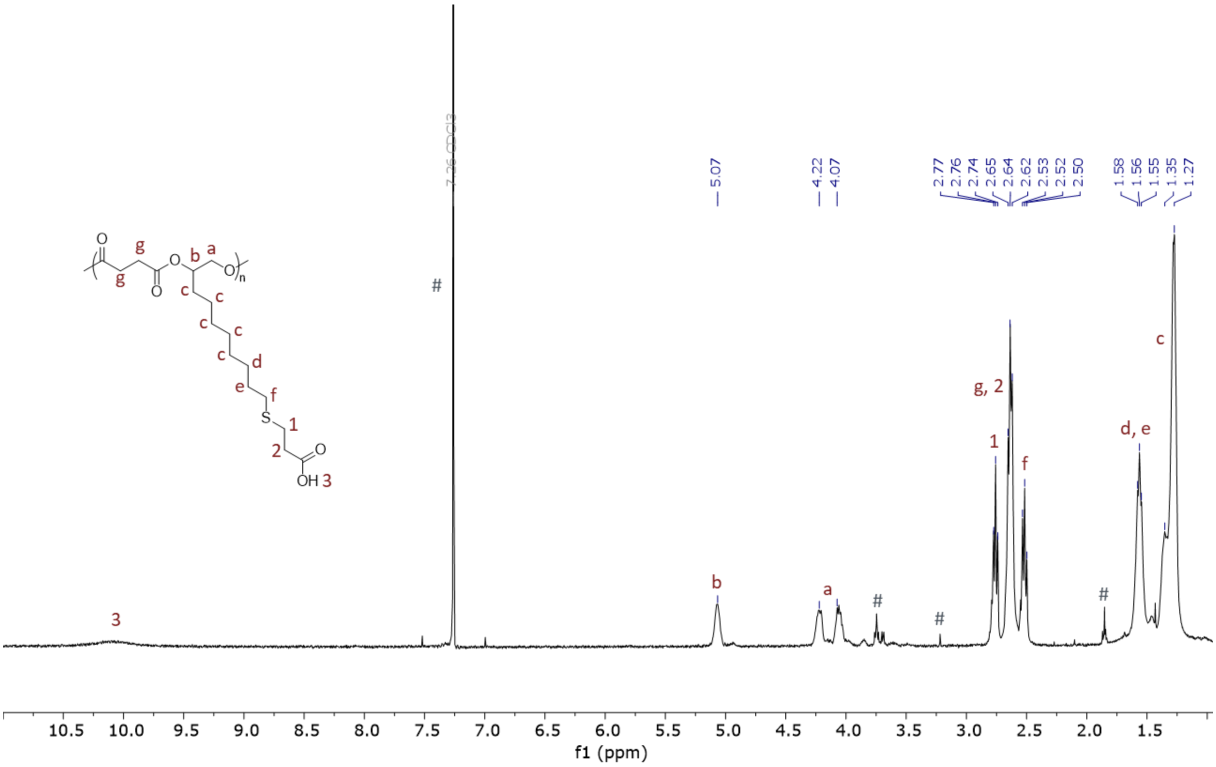

Figure S16. ^{1}H NMR spectrum (400 MHz, $CDCl_3$) of P6-COOH.

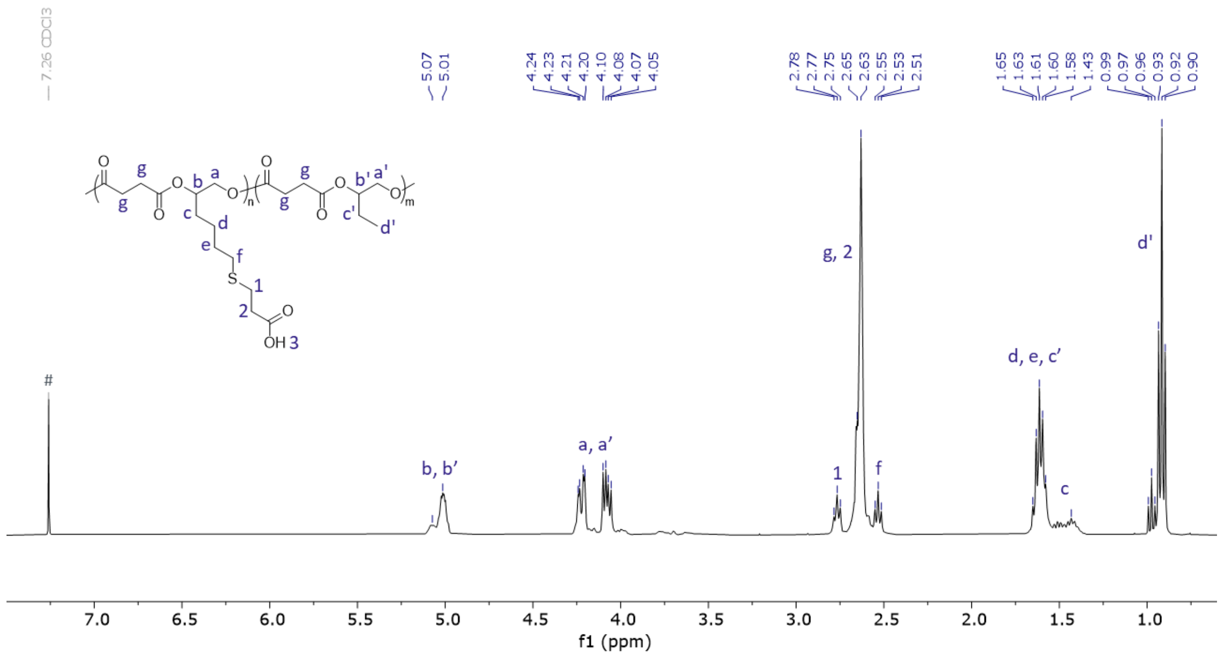

Figure S17. ^{1}H NMR spectrum (400 MHz, $CDCl_3$) of P7-COOH.

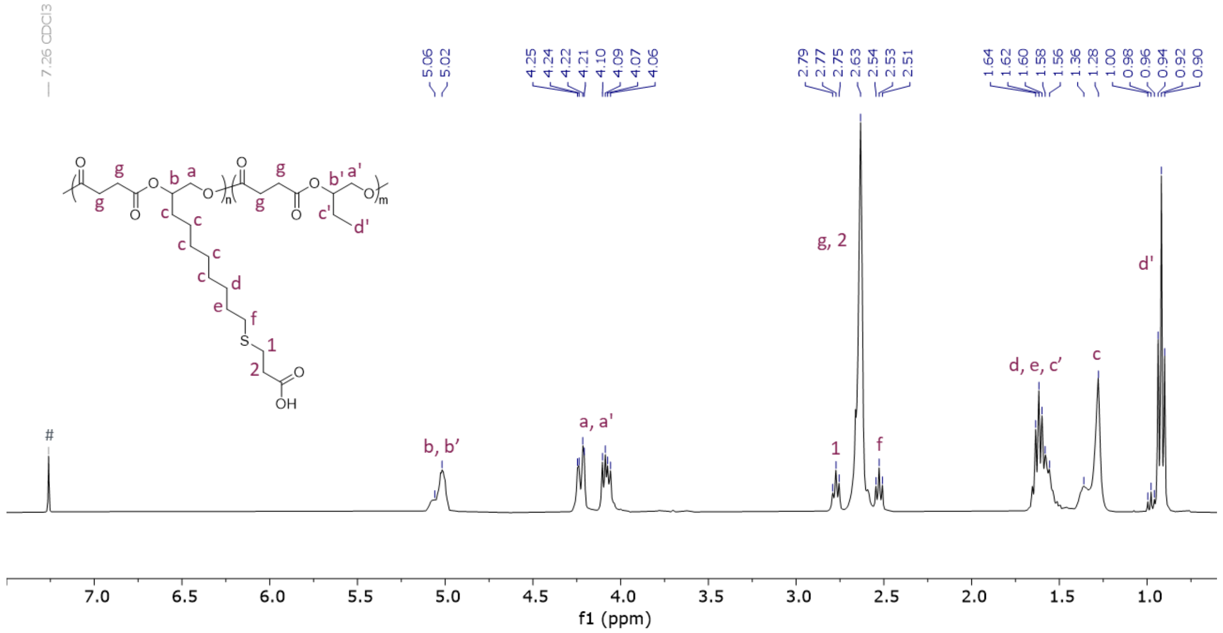

Figure S18. ^{1}H NMR spectrum (400 MHz, $CDCl_3$) of P8-COOH.

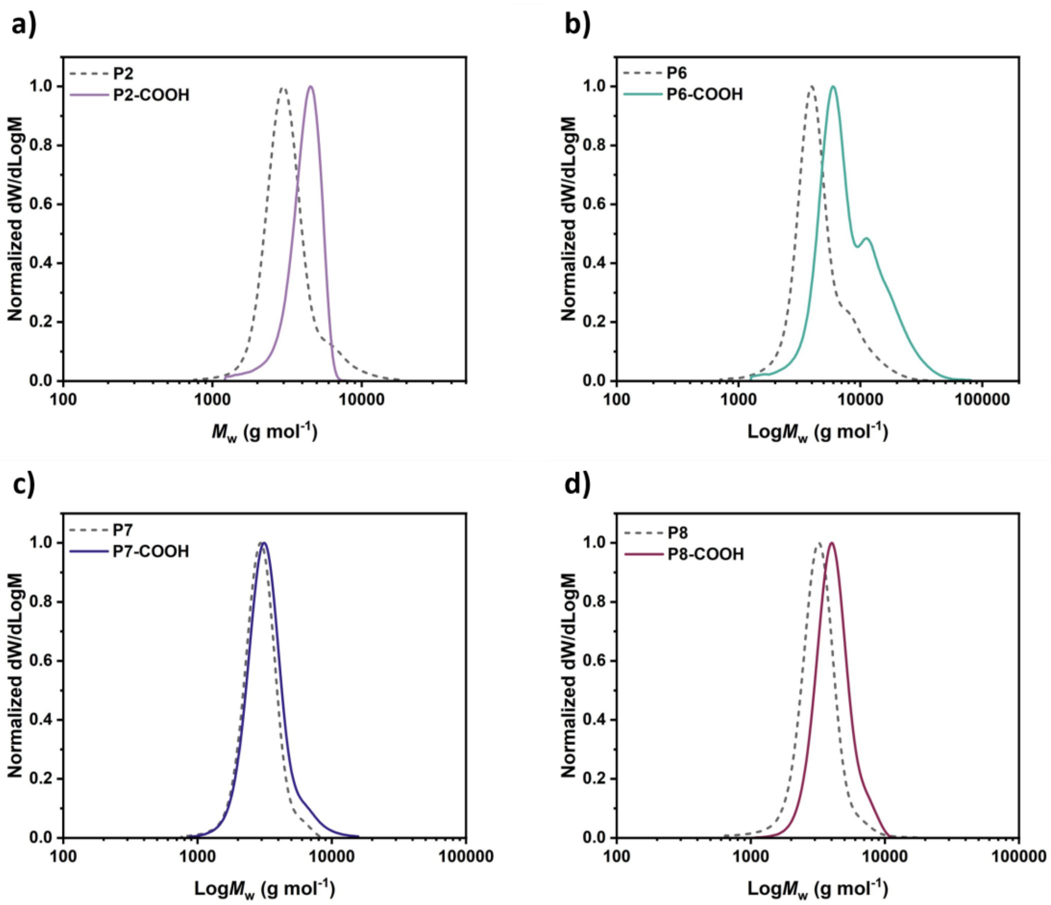

Figure S19. SEC data (in THF) of the copolymers before (dashed line) and after COOH functionalization (solid line) for the copolymers obtained by copolymerization of SA with (a) VHO, (b) vDO, (c) vHO/BO, and (d) vDO/BO.

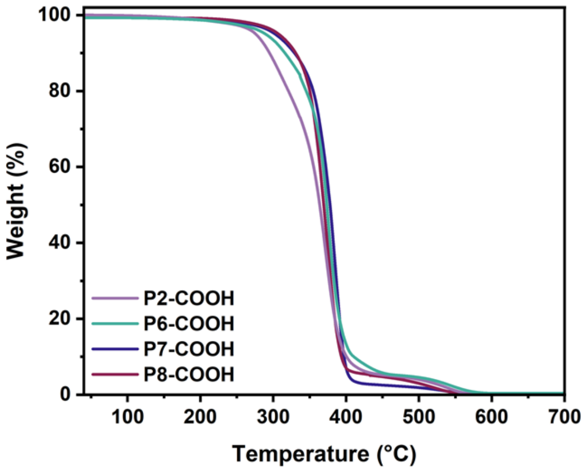

Figure S20. Thermogravimetric curves under air of the COOH-functionalized copolymers.

Table S4. Degradation temperatures of the COOH-functionalized copolymers.

Sample	$T_{d,5\%}$ (°C)	$T_{d,onset}$ (°C)
P2-COOH	276	296
P6-COOH	289	315
P7-COOH	303	350
P8-COOH	307	345

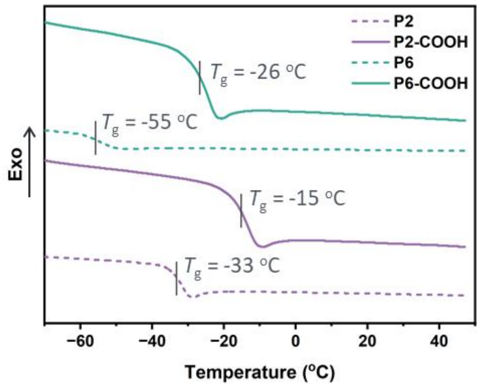

Figure S21. DSC traces of P2 (lilac) and P6 (teal) before (dashed line) and after (solid line) functionalization, showing a single thermal transition corresponding to a glass transition (exo up, normalized to the same heat flow per gram).

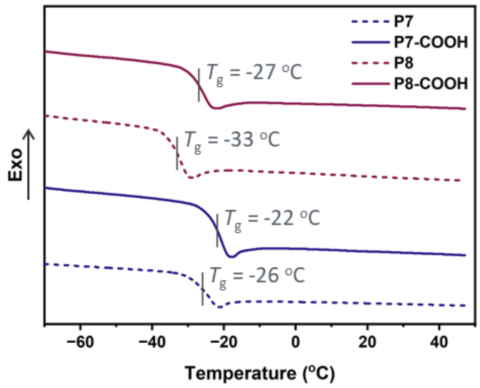

Figure S22 DSC traces of P7 (dark blue) and P8 (plum) before (dashed line) and after (solid line) functionalization, showing a single thermal transition corresponding to a glass transition (exo up, normalized to the same heat flow per gram).

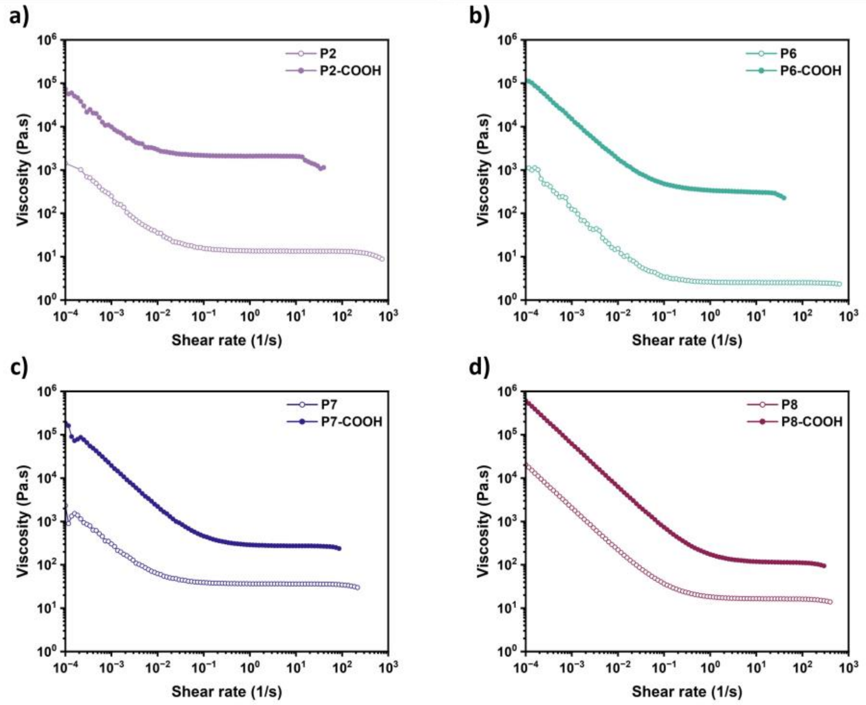

Figure S23. Viscosity as a function of the shear rate measured at 30 °C, for (a) P2, (b) P6, (c) P7, and (d) P8 before (empty circle) and after functionalization (solid circle). The infinite viscosity values were obtained using the cross method.

7. Synthesis and Characterization of Single-Catalyst Polyester Networks

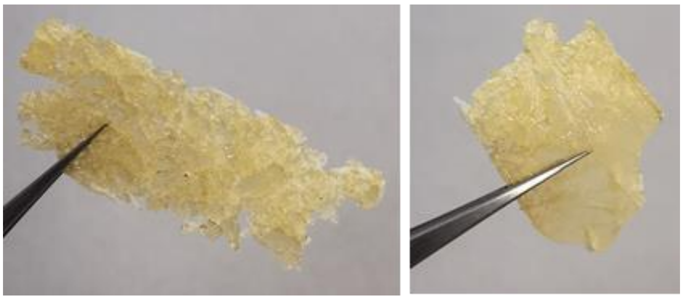

Figure S24. Image of the brittle films obtained using $P_{vDO100\%}$ and $P_{vDO50\%}$ polymers.

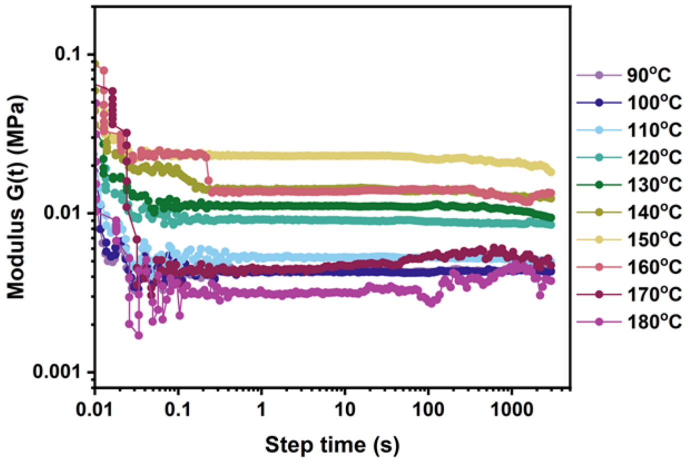

Figure S25. Stress relaxation data for the crosslinked material obtained by reacting the hydroxyl-functionalized copolymer P8-OH (20% functionalization) with DGEBA, using $Zn(Oct)_2$ as the catalyst ([OH]/[epoxy]/[cat]= 1:1:0.05).

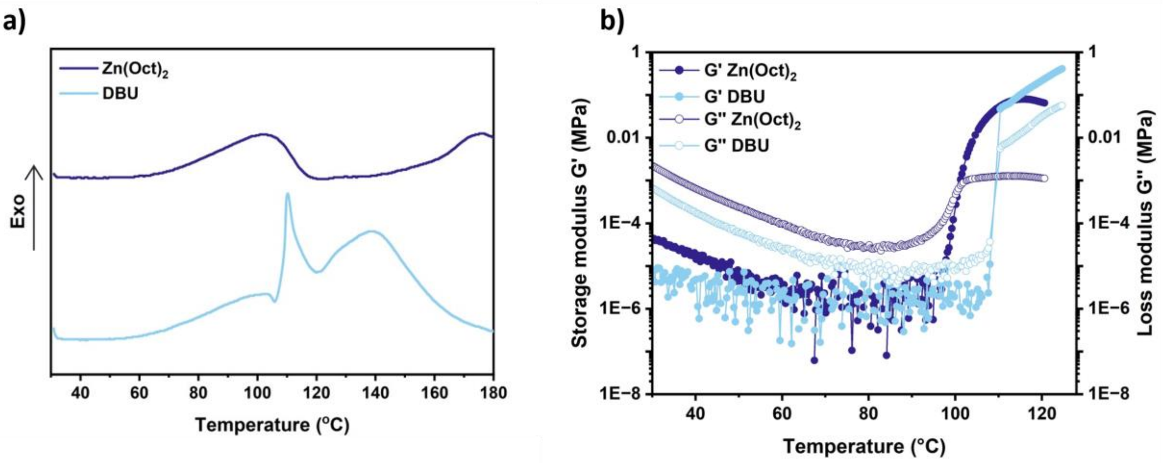

Figure S26. Study of the curing reaction as a function of temperature of mixtures of P8-COOH, DGEBA, and 5 mol% of $Zn(Oct)_2$ (dark blue), or DBU (light blue). (a) DSC data of the first heating cycle (exo up, normalized to the same heat flow per gram), and (b) oscillation temperature ramps.

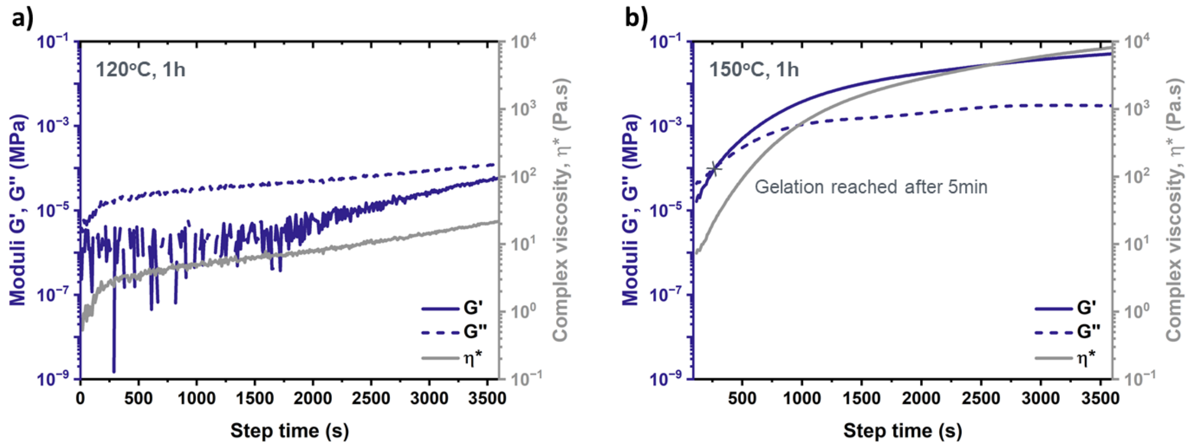

Figure S27. Determination of the gelation point for the curing reaction of a mixture of P8-COOH, DGEBA and 5mol% of $Zn(Oct)_2$. DSC isotherms performed at (a) 120 °C for 1 h, and (b) 150 °C for 1 h.

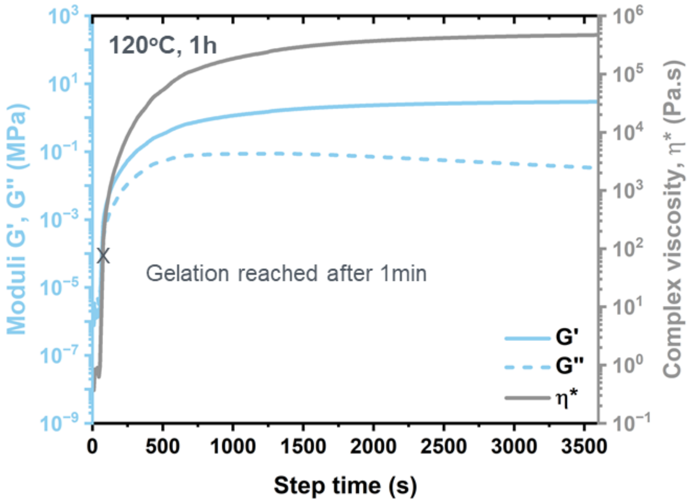

Figure S28. Determination of the gelation point for the curing reaction of a mixture of P8-COOH, DGEBA and 5mol% of DBU. DSC isotherm performed at 120 °C for 1 h.

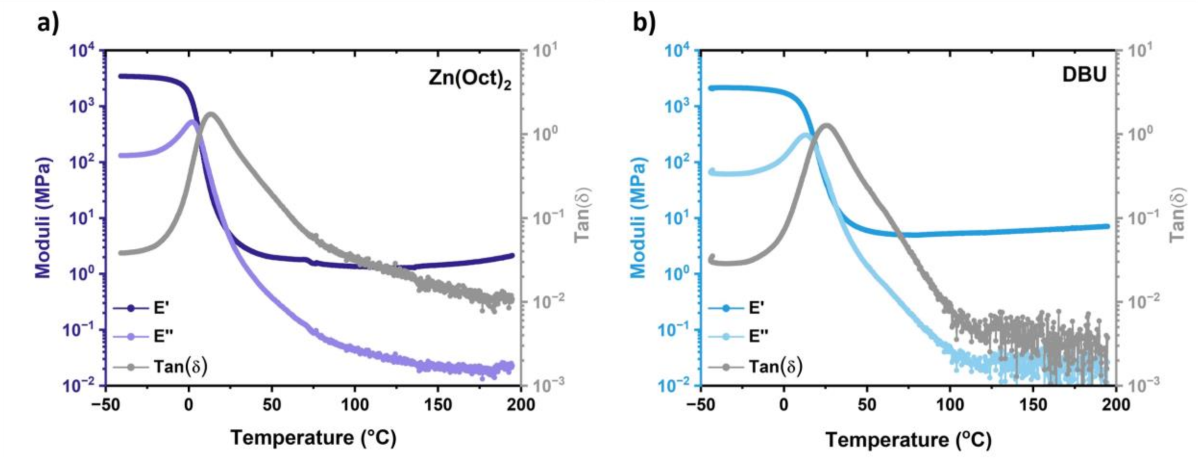

Figure S29. DMA temperature ramp profiles for the two polymer networks cured with (a) $Zn(Oct)_2$, and (b) DBU.

Table S5. Moduli crossover temperature and Tan(δ) peak temperature for the networks cured with $Zn(Oct)_2$ and DBU.

Catalyst	Moduli crossover T (°C)	$Tan(\delta)_{max}$ T (°C)
$Zn(Oct)_2$	6	13
DBU	20	25

Table S6. Summary of the mechanical properties of the P8-COOH/DGEBA cured materials obtained using $Zn(Oct)_2$ or DBU as catalyst.

Catalyst	UTS (MPa)	ε_b (%)	E (MPa)	Toughness (MPa)
$Zn(Oct)_2$	3.05 ± 0.30	238 ± 20	2.46 ± 0.20	3.52 ± 0.43
DBU	14.06 ± 1.59	108 ± 19	287.60 ± 50.27	11.40 ± 2.54

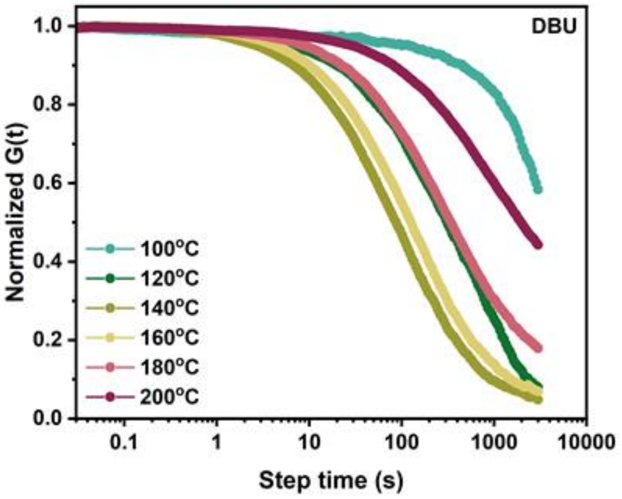

Figure S30. Normalized stress relaxation data for the crosslinked material obtained by curing P8-COOH and DGEBA with DBU, highlighting the catalyst's limited effectiveness in promoting dynamic bond exchange.

8. Synthesis and Characterization of Two-Catalyst Polyester Networks

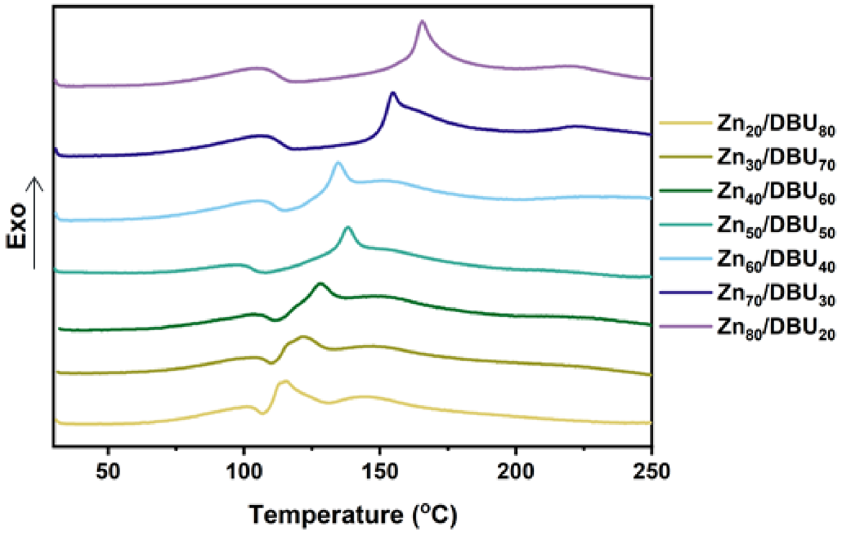

Figure S31. DSC curves of the first heating cycle of the exothermic curing processes of the reaction mixtures containing the 20%-functionalized P8-COOH copolymer, DGEBA, and 5 mol% of different $Zn(Oct)_2$/DBU ratios (exo up, normalized to the same heat flow per gram).

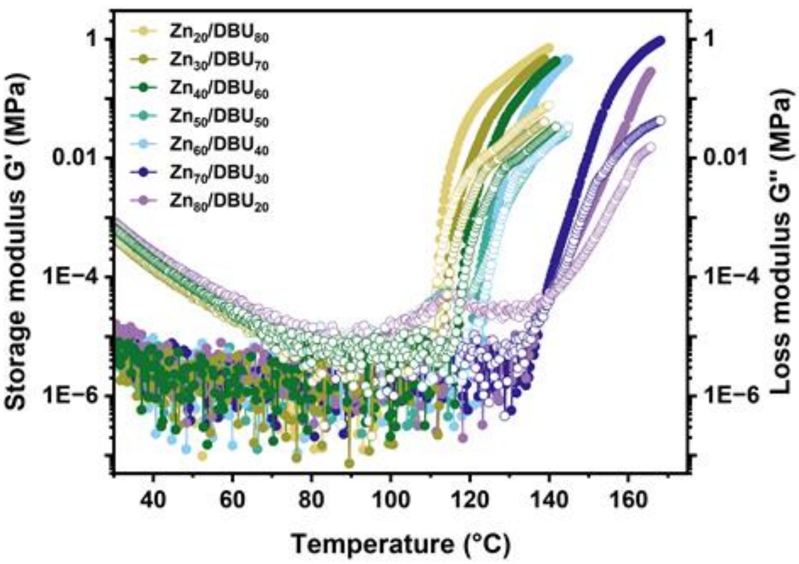

Figure S32. Oscillation temperature ramp of the reaction mixtures containing the 20%-functionalized P8-COOH copolymer, DGEBA, and different $Zn(Oct)_2$/DBU ratios. G' and G'' were measured as a function of temperature and the gelation temperature (T_{gel}) was determined at the moduli crossover point. T_{gel} was taken as the minimum temperature required for curing the polymer networks.

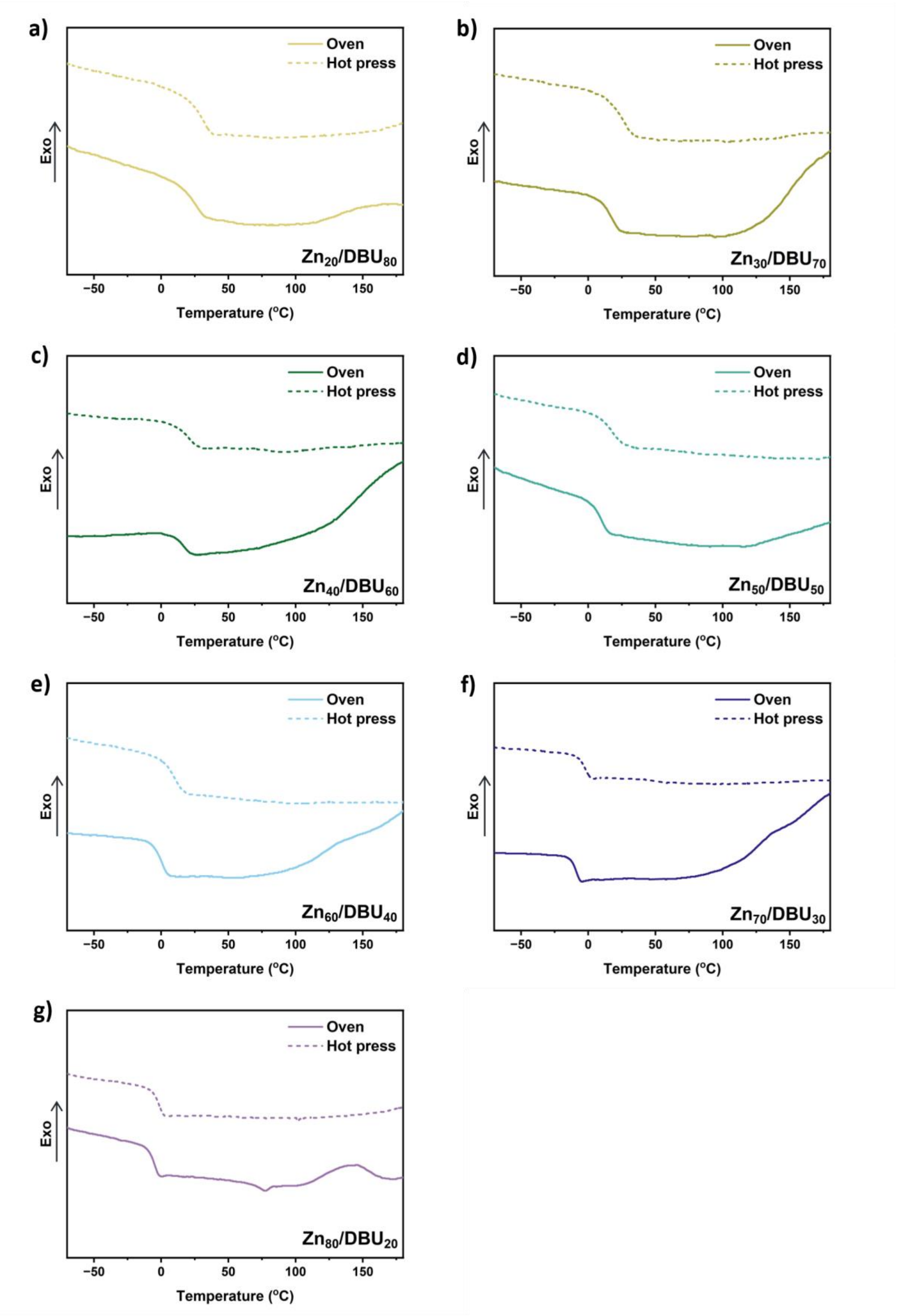

Figure S33. Determination of the curing conditions for the networks obtained by varying the $Zn(Oct)_2$/DBU ratio. DSC curves of the networks after the initial curing treatment in the oven at 120 °C for 1 h (solid line), and after the hot press treatment (dashed line). The disappearance of the exothermic peak in the dashed line indicates complete curing. Data are shown for P8-COOH/DGEBA mixtures with $Zn(Oct)_2$/DBU ratios of (a) 20:80, (b) 30:70, (c) 40:60, (d) 50:50, (e) 60:40, (f) 70:30, and (g) 80:20.

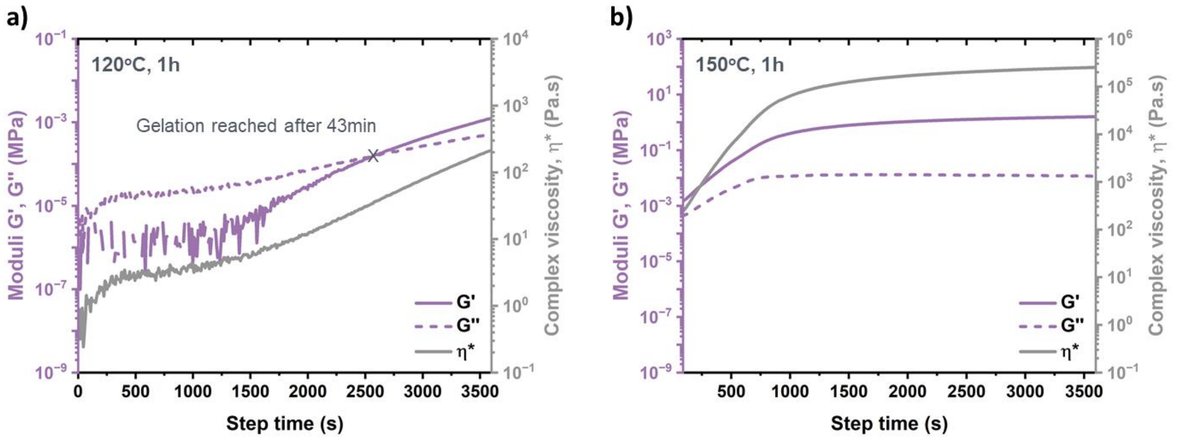

Figure S34. Determination of the gelation point for the curing reaction of a P8-COOH/DGEBA mixture containing Zn(Oct)$_2$:DBU= 80:20. DSC isotherms performed at (a) 120 °C for 1 h, and (b) 150 °C for 1 h.

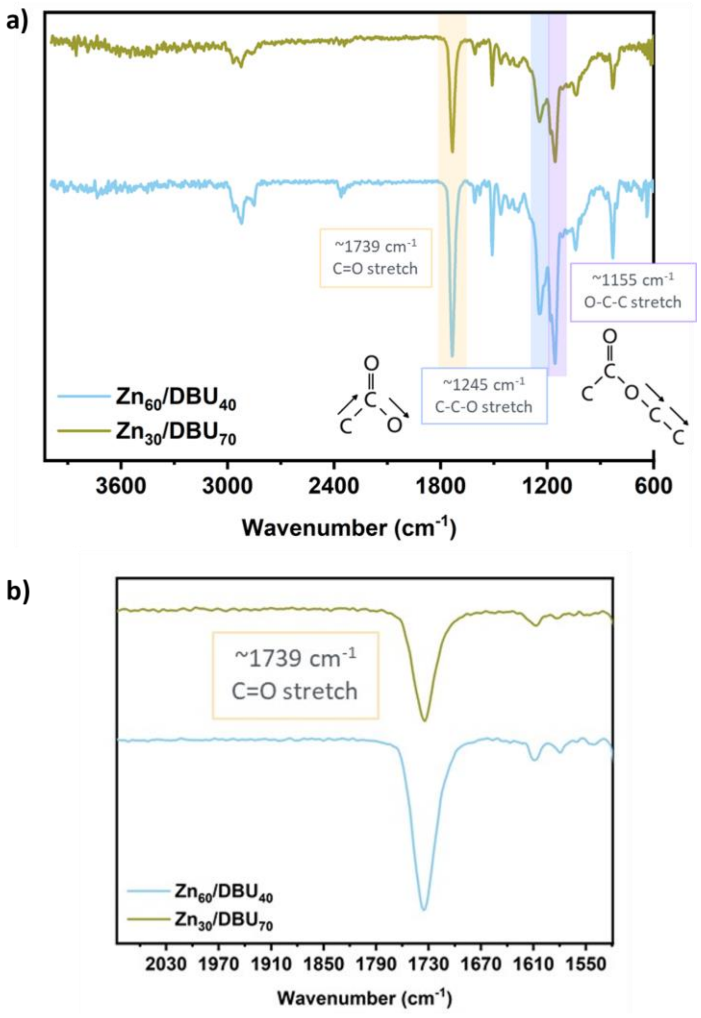

Figure S35. FTIR analysis of dry films. (a) FTIR spectra of the Zn$_{60}$/DBU$_{40}$ and Zn$_{30}$/DBU$_{70}$ networks, and (b) enlarged view of the wavenumber region corresponding to the C=O stretch, highlighting only the peak associated with the ester C=O stretch.

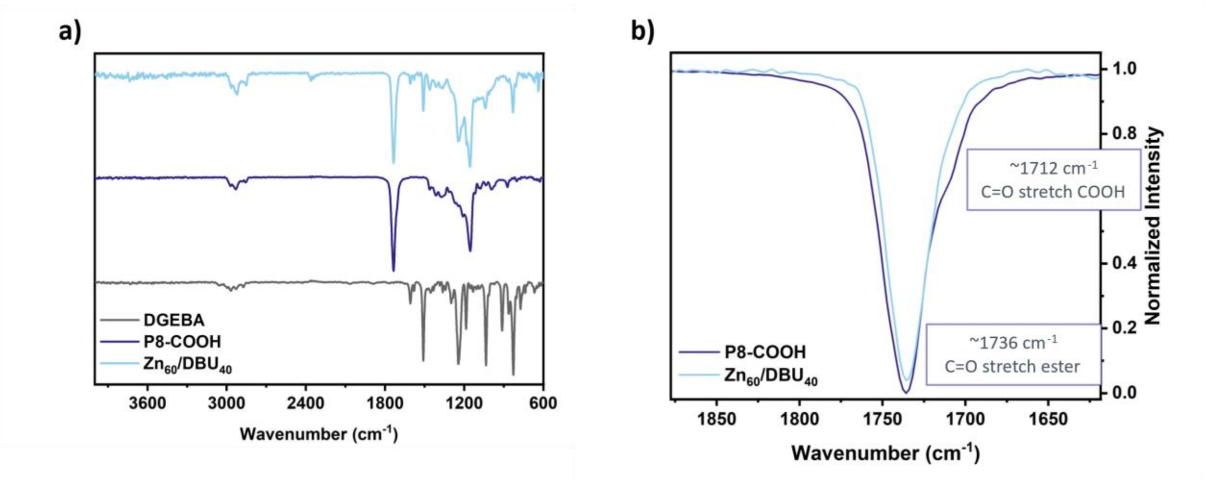

Figure S36. FTIR analysis of dry films. (a) FTIR spectra of the Zn_{60}/DBU_{40} film, the COOH-containing polymer P8-COOH, and the epoxy crosslinker DGEBA. (b) enlarged view of the wavenumber region corresponding to the C=O stretch, highlighting the presence of a shoulder in the case of the polymer, corresponding to the COOH C=O stretch.

Table S7. Thermal properties of the crosslinked materials obtained by varying the $Zn(Oct)_2$/DBU ratio in the reaction mixture.

$Zn(Oct)_2$:DBU	T_g (°C)	$T_{d,onset}/T_{d,5\%}$ (°C)
20:80	28	310/280
30:70	24	297/269
40:60	20	294/266
50:50	13	284/272
60:40	8	291/263
70:30	-3	280/268
80:20	-2	280/269

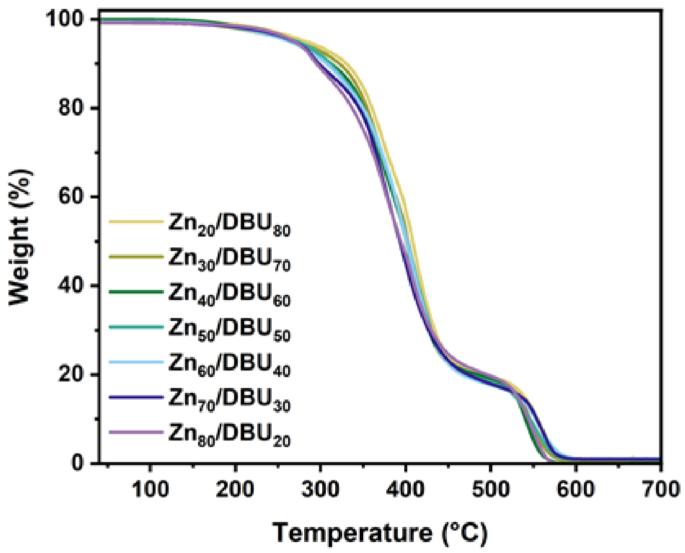

Figure S37. Thermogravimetric curves under air of the crosslinked materials obtained my curing P8-COOH and DGEBA with different $Zn(Oct)_2$/DBU ratios.

Table S8. Swelling experiments performed in THF for the crosslinked materials obtained my curing P8-COOH and DGEBA with different Zn(Oct)$_2$/DBU ratios.

Zn(Oct)$_2$:DBU	Swelling index (%)	Gel content (%)
30:70	73 ± 2	98 ± 1
50:50	69 ± 1	97 ± 1
60:40	100 ± 4	93 ± 0
70:30	308 ± 4	61 ± 2

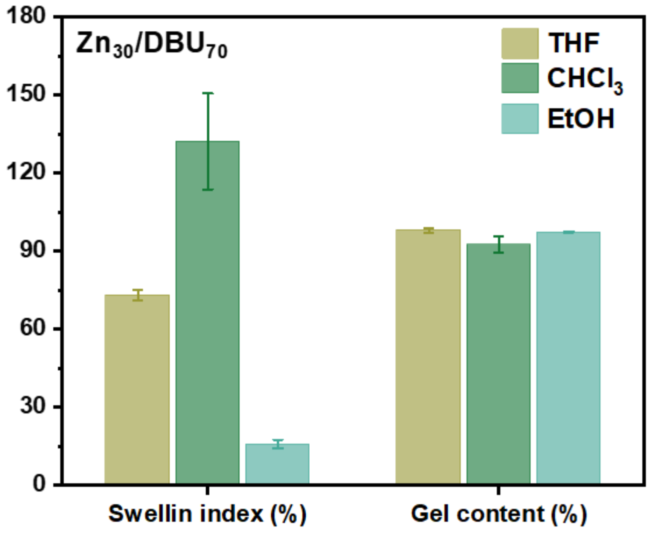

Figure S38. Swelling index and gel content calculated after swelling the Zn(Oct)$_2$/DBU=30:70 polymer network in THF, chloroform, and ethanol for 24 h and drying for 96 h.

Table S9. Mechanical properties of the crosslinked materials obtained by curing P8-COOH and DGEBA with different Zn(Oct)$_2$/DBU ratios.

Zn(Oct)$_2$:DBU	ε_b (%)	UTS (MPa)	E (MPa)	Toughness (MPa)
30:70	89 ± 8	23.28 ± 1.46	418.19± 13.75	16.73 ± 1.61
50:50	121 ± 16	17.17 ± 1.69	306.30 ± 36.15	13.98 ± 2.55
60:40	221 ± 18	8.33 ± 1.34	30.99 ± 1.24	8.91 ± 1.54
70:30	421 ± 50	0.33 ± 0.05	0.26 ± 0.03	0.65 ± 0.13

9. Study of the Dynamic Bond Exchange in the Two-Catalyst Polyester Networks

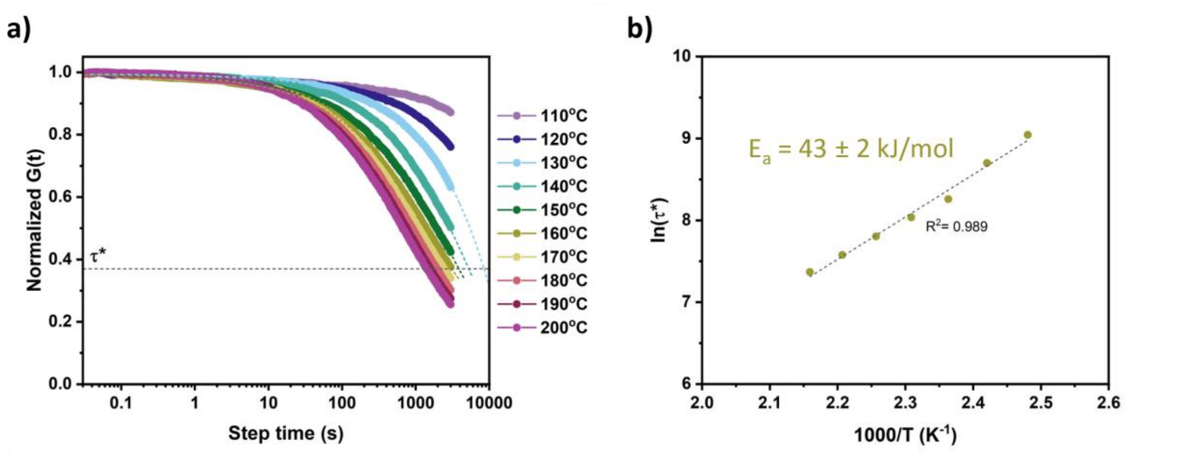

Figure S39. Stress relaxation data for the crosslinked material containing $Zn(Oct)_2$/DBU= 30:70. (a) Normalized stress relaxation data, and (b) plot of ln(τ*) vs 1000/T with a linear fit to extract the activation energy.

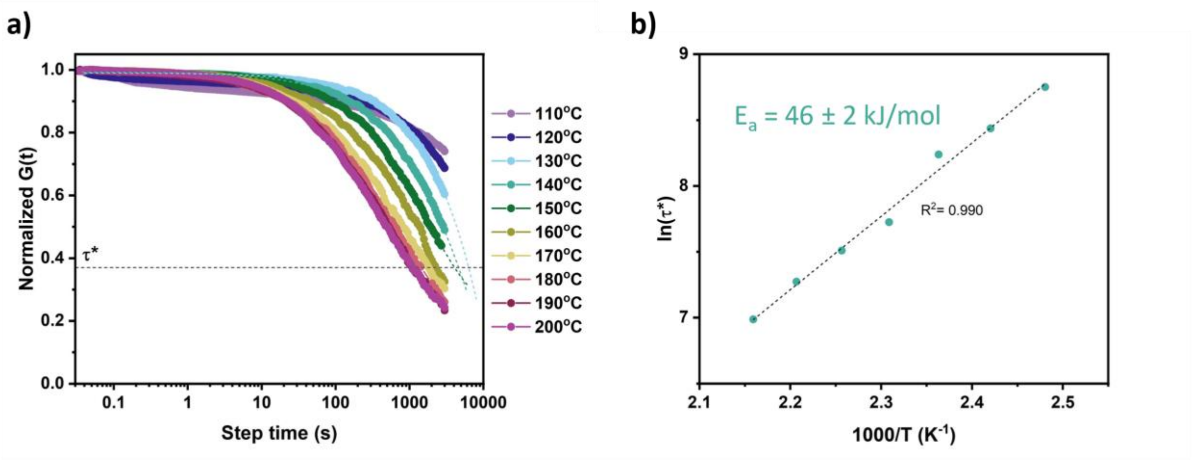

Figure S40. Stress relaxation data for the crosslinked material containing $Zn(Oct)_2$/DBU= 50:50. (a) Normalized stress relaxation data, and (b) plot of ln(τ*) vs 1000/T with a linear fit to extract the activation energy.

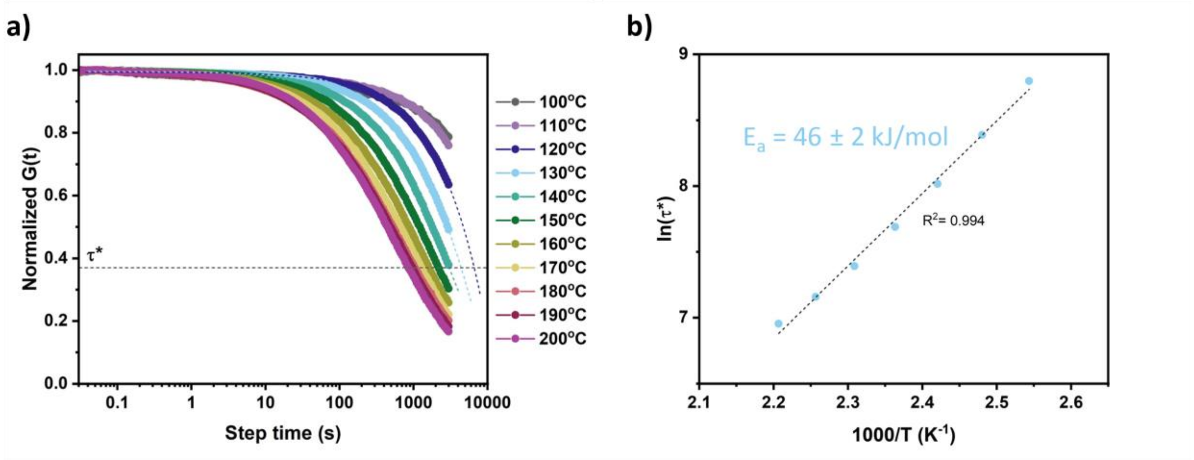

Figure S41. Stress relaxation data for the crosslinked material containing $Zn(Oct)_2$/DBU= 60:40. (a) Normalized stress relaxation data, and (b) plot of ln(τ*) vs 1000/T with a linear fit to extract the activation energy.

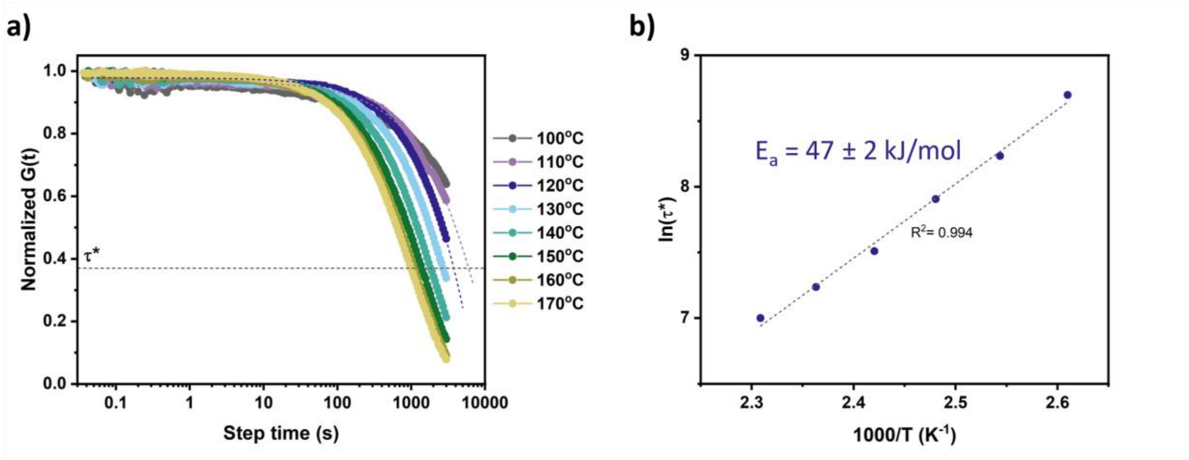

Figure S42. Stress relaxation data for the crosslinked material containing $Zn(Oct)_2$/DBU= 70:30. (a) Normalized stress relaxation data, and (b) plot of ln(τ*) vs 1000/T with a linear fit to extract the activation energy.

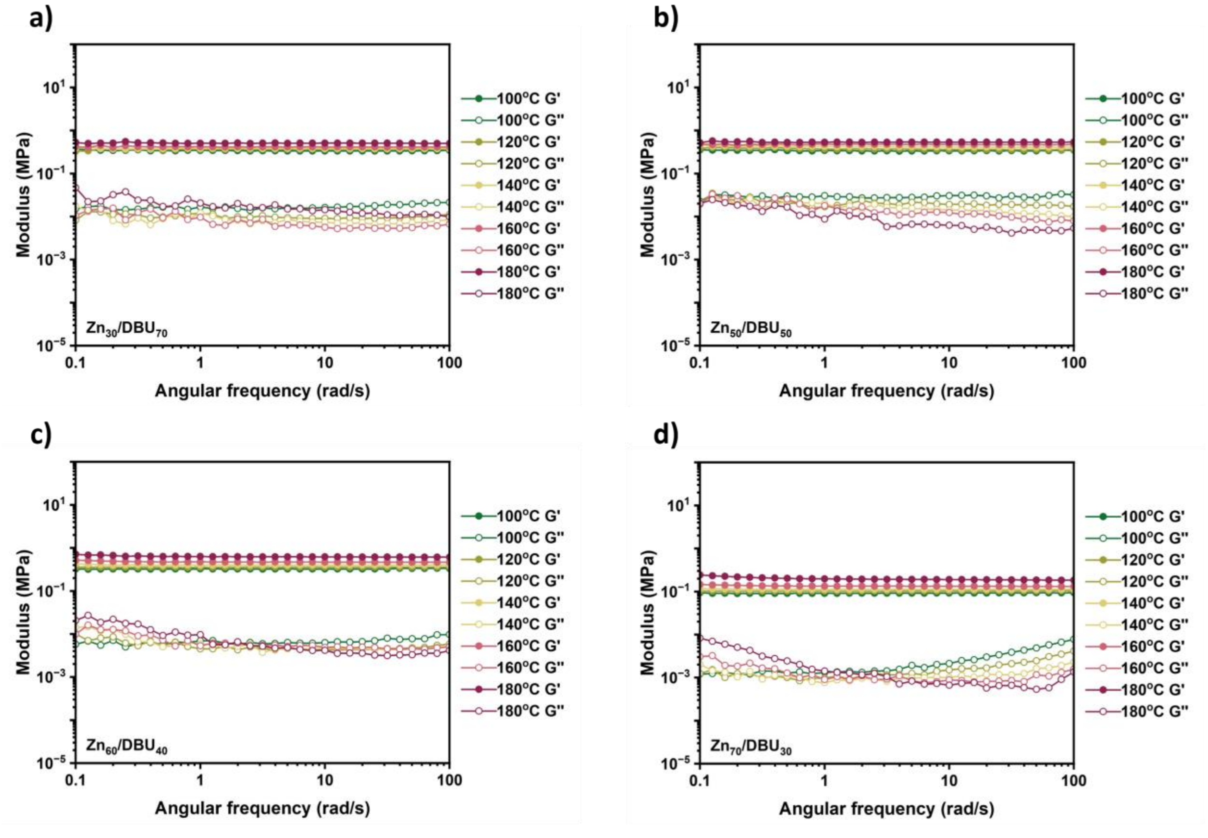

Figure S43. Frequency sweep experiments performed between 100 °C and 180 °C for (a) Zn_{30}/DBU_{70}, (b) Zn_{50}/DBU_{50}, (c) Zn_{60}/DBU_{40}, and (d) Zn_{70}/DBU_{30}.

10. Dimensional Stability, Reproducibility and Reprocessability of the Two-Catalyst Polyester Networks

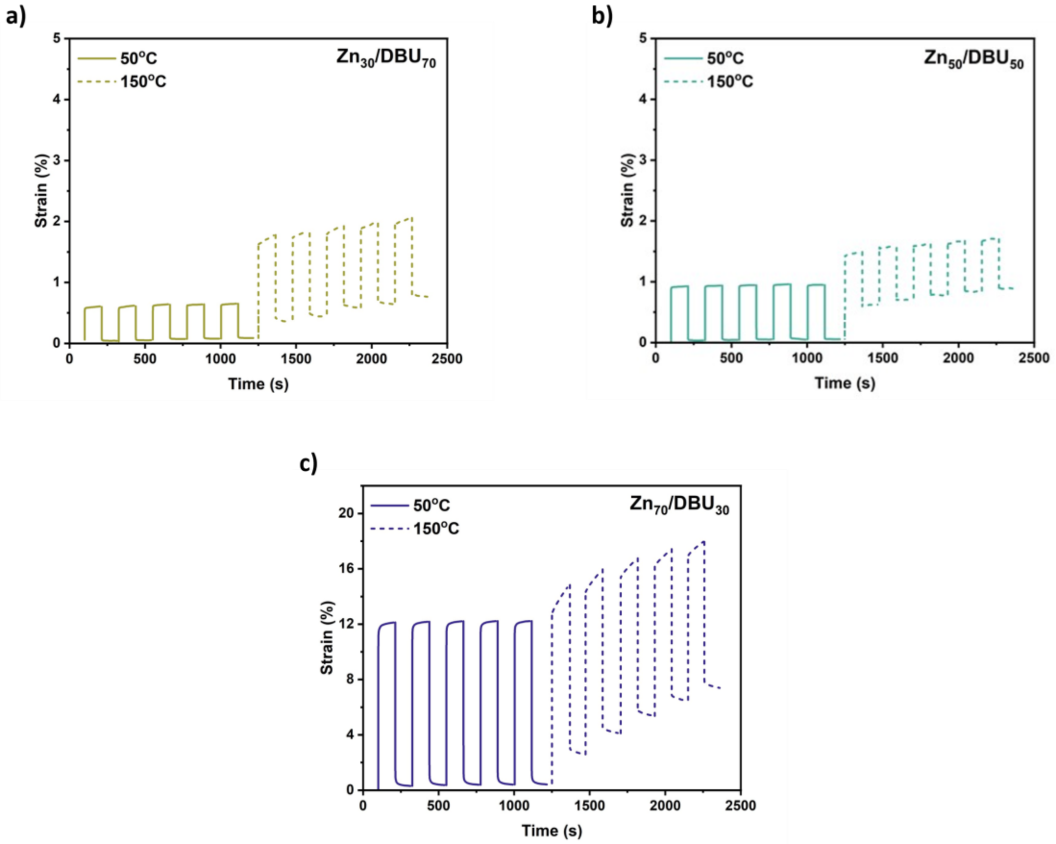

Figure S44. Cyclic creep recovery experiments performed at 50 °C (solid line) and 150 °C (dashed line) on the (a) Zn_{30}/DBU_{70}, (b) Zn_{50}/DBU_{50}, and (c) Zn_{70}/DBU_{30} networks.

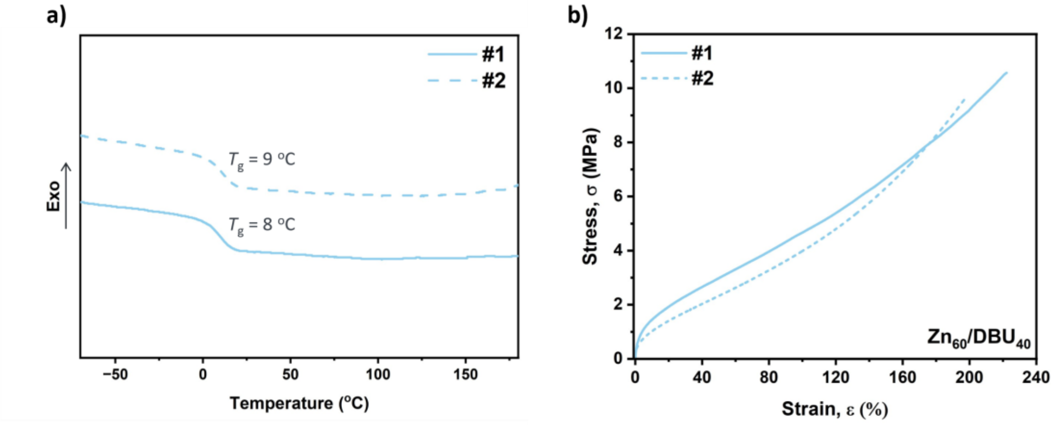

Figure S45. Comparison of the thermomechanical properties between the two individual networks synthesized by curing P8-COOH, DGEBA and $Zn(Oct)_2$/DBU=60:40. (a) DSC data (exo up, normalized to the same heat flow per gram), and (b) representative stress–strain curves (10 mm min^{-1} extension rate).

Table S10. Mechanical properties of the two separate networks obtained by curing P8-COOH, DGEBA and $Zn(Oct)_2$/DBU=60:40.

Sample	ε_b (%)	UTS (MPa)	E (MPa)	Toughness (MPa)
#1	221 ± 18	8.33 ± 1.34	30.99 ± 1.24	8.91 ± 1.54
#2	202 ± 14	9.79 ± 1.64	29.15 ± 1.99	9.26 ± 2.22

Table S11. Mechanical properties of the Zn_{60}/DBU_{40} network before and after two recycling cycles.

Sample	ε_b (%)	UTS (MPa)	E (MPa)	Toughness (MPa)
Original Network	221 ± 18	8.33 ± 1.34	30.99 ± 1.24	8.91 ± 1.54
Recycledx1 1	132 ± 34	9.24 ± 2.08	35.08 ± 9.85	6.76 ± 2.47
Recycledx2	136 ± 38	9.71 ± 1.27	52.93 ± 10.10	5.13 ± 1.07

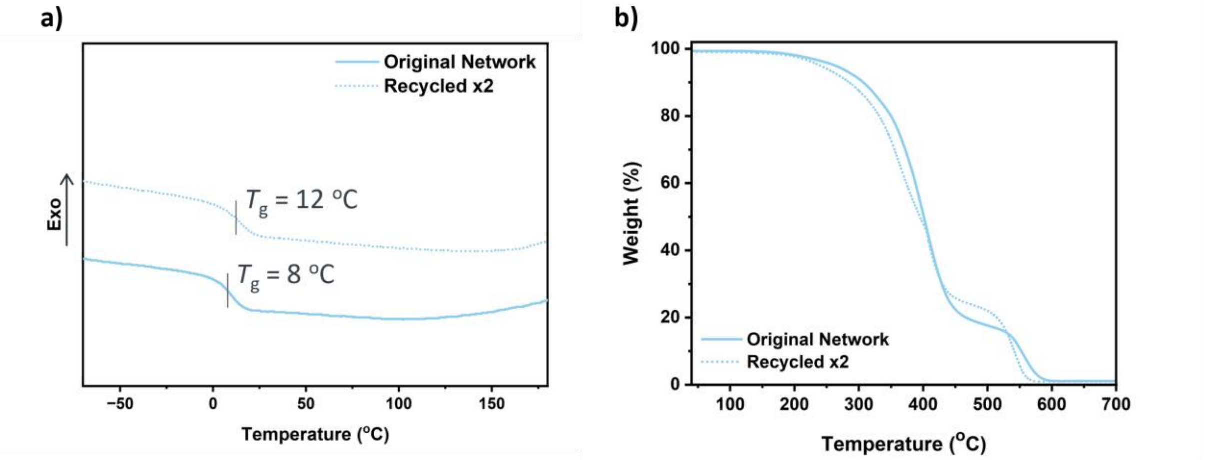

Figure S46. Thermal properties of the Zn_{60}/DBU_{40} network before (solid line) and after the second reprocessing cycle (dashed line). (a) DSC data (exo up, normalized to the same heat flow per gram), and (b) thermogravimetric curves under air.

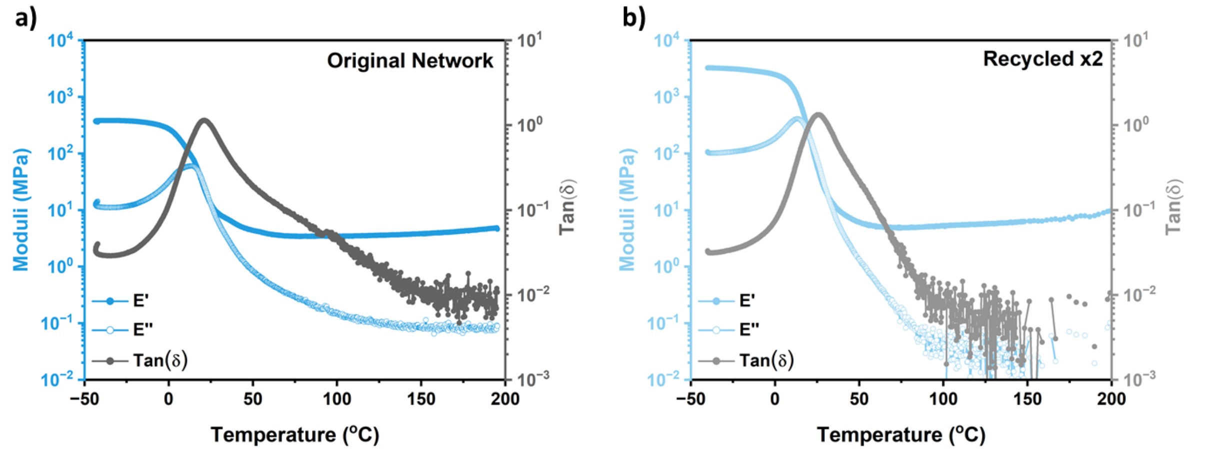

Figure S47. DMA temperature ramp profiles for (a) the original network, and (b) the network after two recycling cycles.

Table S12. Moduli crossover temperature and Tan(δ) peak temperature for the original and twice recycled networks.

Sample	Moduli crossover T (°C)	$Tan(\delta)_{max}$ T (°C)
Original Network	15	20
Recycled x2	20	26

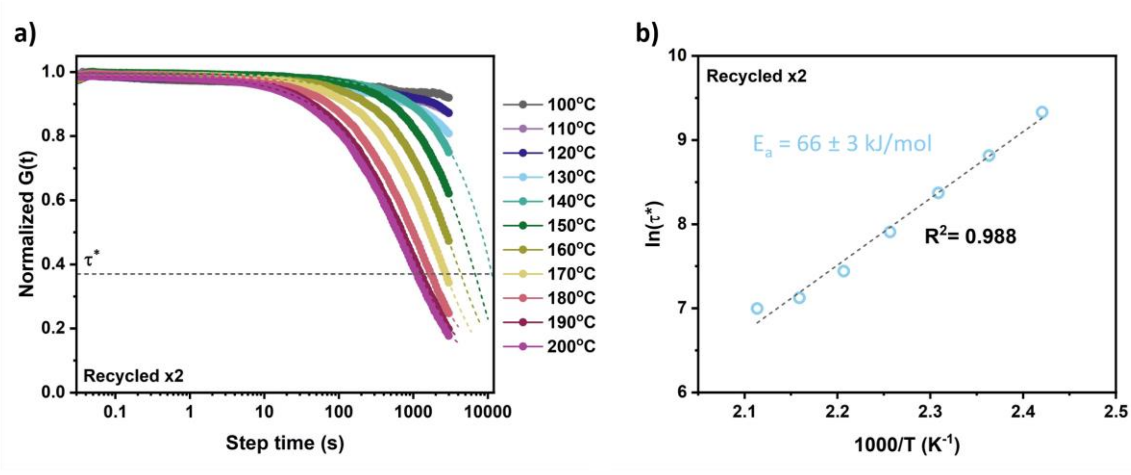

Figure S48. Stress relaxation data for the crosslinked material containing $Zn(Oct)_2$/DBU= 60:40 after two recycling cycles. (a) Normalized stress relaxation data, and (b) plot of ln(τ*) vs 1000/T with a linear fit to extract the activation energy.

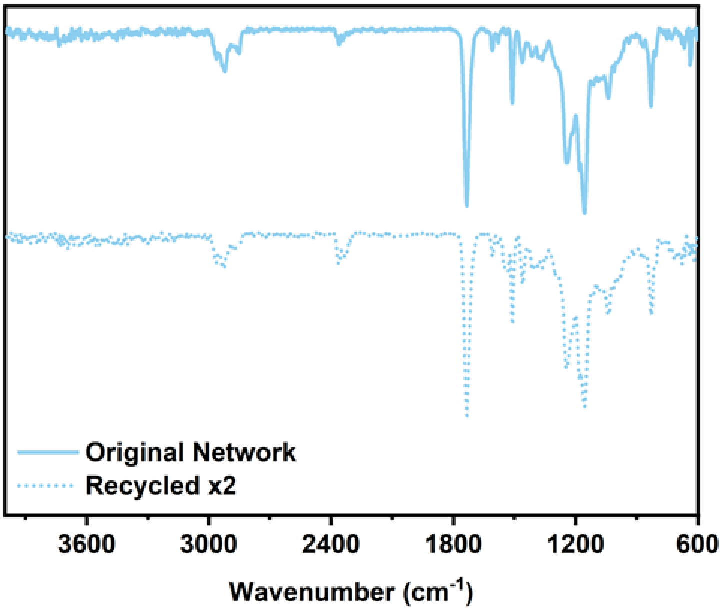

Figure S49. FTIR spectra of the Zn_{60}/DBU_{40} network before (solid line) and after the second reprocessing cycle (dotted line).
